# High fat / high cholesterol diet does not provoke atherosclerosis in the ω3-and ω6-polyunsaturated fatty acid synthesis–inactivated Δ6-fatty acid desaturase–deficient mouse

**DOI:** 10.1016/j.molmet.2021.101335

**Published:** 2021-09-14

**Authors:** Wilhelm Stoffel, Erika Binczek, Inga Schmidt-Soltau, Susanne Brodesser, Ina Wegner

**Affiliations:** 1 Laboratory of Molecular Neuroscience, Institute of Biochemistry, University of Cologne, 50931, Cologne, Germany; 2 Center for Molecular Medicine (CMMC), Faculty of Medicine, University of Cologne, 50931, Cologne, Germany; 3 Cluster of Excellence, Cellular Stress Response in Aging-Related Diseases (CECAD), University of Cologne, Germany

**Keywords:** PUFA synthesis deficiency, Atherogenic nutrition, Hepatic lipid metabolism, Dyslipoproteinemia, Atherosclerotic lesions

## Abstract

**Objective:**

An increased ω6/ω3-polyunsaturated fatty acid ratio in the current Western diet is regarded as a critical epigenetic nutritional factor in the pathogenesis of several human lifestyle diseases, metabolic syndrome, cardiovascular disease, the central nervous system and the female and male reproductive systems. The impact of nutrient ω3-and ω6-PUFAs in the pathogenesis of dyslipoproteinemia and atherosclerosis has been a topic of intense efforts for several decades. Cellular homeostasis of the ω3-and ω6- PUFA pool is maintained by the synthesis of ω3-and ω6-PUFAs from essential fatty acids (EFA) (linoleic and α-linolenic acid) and their dietary supply.

In this study, we used the auxotrophic Δ6-fatty acid desaturase- (FADS2) deficient mouse (*fads2*−/−), an unbiased model congenial for stringent feeding experiments, to investigate the molecular basis of the proposed protective role of dietary ω3-and ω6-PUFAs (Western diet) in the pathogenesis of multifactorial dyslipoproteinemia and atherosclerosis. We focused on the metabolic axis—liver endoplasmic reticulum (ER), serum lipoprotein system (Lp) and aorta vessel wall. Furthermore, we addressed the impact of the inactivated *fads2*-locus with inactivated PUFA synthesis on the development and progression of extended atherosclerosis in two different mouse mutants with disrupted cholesterol homeostasis, using the *apoe*−/− and *ldlr*−/− mutants and the *fads2*−/− *x apoe*−/− and *fads2*−/− *x ldlr*−/− double mutants.

**Methods:**

Cohorts of +/+ and *fads2*−/− mice underwent two long-term dietary regimens: a) a PUFA-free standard chow diet containing only EFAs, essential for viability, and b) a high fat/high cholesterol (HFHC) diet, a mimicry of the human atherogenic “Western” diet. c) To study the molecular impact of PUFA synthesis deficiency on the development and progression of atherosclerosis in the hypercholesterolemic *apoe*−/− and *ldlr*−/− mouse models fed PUFA-free regular and sustained HFHC diets, we generated the *fads2*−/− *x apoe*−/− and the *fads2*−/− *x ldlr*−/− double knockout mutants. We assessed essential molecular, biochemical and cell biological links between the diet-induced modified lipidomes of the membrane systems of the endoplasmic reticulum/Golgi complex, the site of lipid synthesis, the PL monolayer and neutral lipid core of LD and serum-Lp profiles and cellular reactions in the aortic wall.

**Results:**

ω3-and ω6-PUFA synthesis deficiency in the *fads2*−/− mouse causes a) hypocholesterolemia and hypotriglyceridemia, b) dyslipoproteinemia with a shift of high-density lipoprotein (HDL) to very low-density lipoprotein (VLDL)-enriched Lp-pattern and c) altered liver lipid droplet structures. d) Long-term HFHC diet does not trigger atherosclerotic plaque formation in the aortic arc, the thoracic and abdominal aorta of PUFA-deficient *fads2*−/− mice. Inactivation of the *fads2*−/− locus, abolishing systemic PUFA synthesis in the *fads2*−/− *x apoe*−/− and *fads2*−/− *x ldlr*−/− double knockout mouse lines.

**Conclusions:**

Deficiency of ω3-and ω6-PUFA in the *fads2*−/− mutant perturbs liver lipid metabolism, causes hypocholesterolemia and hypotriglyceridemia and renders the *fads2*−/− mutant resistant to sustained atherogenic HFHC diet. Neither PUFA-free regular nor long-term HFHC-diet impacts the *apoe*- and LDL-receptor deficiency–provoked hypercholesterolemia and atherosclerotic plaque formation, size and distribution in the aorta. Our study strongly suggests that the absence of PUFAs as highly vulnerable chemical targets of autoxidation attenuates inflammatory responses and the formation of atherosclerotic lesions.

The cumulative data and insight into the molecular basis of the pleiotropic functions of PUFAs challenge a differentiated view of PUFAs as culprits or benefactors during a lifespan, pivotal for legitimate dietary recommendations.

## Highlights

• ω3-and ω6-PUFA synthesis deficiency in the auxotrophic fads2−/− mouse.
• Perturbs liver membrane lipidomes and lipid metabolism
• Remodels the lipid droplet- and serum lipoprotein-systems
• Prevents PUFA-derived peroxidation products, protein modification, and inflammation
• Protects from high fat/high cholesterol (“Western diet”) that promotes atherosclerosis

## Abbreviations

LD: lipid droplet
Lp: lipoprotein
TAG: triacylglycerol
PC: phosphatidyl-choline
PE: phosphatidyl-ethanolamine
PI: phosphatidyl-inositol
PS: phosphatidyl-serine
C: cholesterol
CE: cholesterol-ester
SM: sphingomyelin
PLIN1: Perilipin 1
PLIN2: Adipophilin, Perilipin 2
ADRP: Adipose Differentiation-Related Protein
PLIN3: Perilipin-3
Tip47: tail interacting protein of 47 kD
ATGL: adipocyte triacylglycerol lipase
DGAT: diacylglycerol-*O*-acyltransferase1
EFA: essential fatty acids
PUFA: polyunsaturated fatty acids
HFHC: high fat high cholesterol
TG: triglyceride
PL: phospholipids
NL: neutral lipids
fads: fatty acid desaturase
HDL: high density lipoprotein
VLDL: very low-density lipoprotein
ppar: peroxisome proliferator-activated receptor α, γ,γc1
srebp1: sterol regulatory element-binding protein-1
scd1: stearoyl-CoA desaturase 1
fas: fatty acid synthase
cyp4a: cytochrome P450 4a

## 1 Introduction

Essential fatty acids (EFA) linoleic and α-linolenic acid and derived long-chain (LC) and very long-chain (VLC) ω3-and ω6-polyunsaturated fatty acids (PUFAs) are pivotal structural elements in the architecture of all mammalian membrane systems and precursors of structurally and functionally diverse signaling molecules operative in pathways regulating lipid and energy metabolism [1]. Homeostasis of the cellular pool of ω3-and ω6- PUFAs is maintained by dietary supply and the transformation of EFAs to LC- and VLC-ω3- and ω6-PUFAs in an orchestrated sequence at the tetrameric chain-elongation and trimeric desaturase complexes in the endoplasmic reticulum, the dominant site of PUFA synthesis.

A variety of genetically conditioned dyslipoproteinemias, notably hypercholesterolemia and hypertriglyceridemia, play a crucial role in the pathogenesis of fatal atherosclerosis in coronary heart disease (CHD). For understanding the mechanism underlying the molecular pathogenesis, two genetically defined murine models have been described: the homozygous *apoe*−/− and *ldlr*−/− mouse mutants. They share the same or similar characteristic pathogenetic features leading to atherosclerosis in humans, despite their different genetic background.

Increased consumption of ω6-PUFAs in Western diets has led to a considerable imbalance of the ω3/ω6-PUFA ratio over the last several decades and is regarded as critical in the pathogenesis of several human genetic and lifestyle diseases: cardiovascular disease, dyslipoproteinemia, obesity, neurodegenerative diseases and infertility. The role of nutrient ω3-and ω6-PUFAs as epigenetic factors in the pathogenesis of atherosclerosis has been assessed in numerous nutritional trials [2,3,5,6]. These studies led to dietary recommendations from the American Heart Association and FAO/WHO expert consultation on fats and fatty acids in human nutrition rating the impact of ω3-and ω6- PUFAs on physiological and pathophysiological conditions at the two highest levels of strength of evidence, “Convincing” and “Probable,” to “conclude that ω6-and ω3-PUFAs affect major health and disease outcomes.” ω3-PUFAs are “convincing” in lowering fatal CHD events and ω6-PUFAs are “probable” for lowering risk of metabolic syndrome components and diabetes [2].

However, due to the complexity and critical parameters, the validity of the outcome of the numerous dietary studies in different species (including humans) has provided only limited insight into the molecular basis of the underlying pathophysiology. Two recent extensive, contradictory reviews of randomized interventional trials [3,4] witness the current need for strategies for the molecular analysis of well-designed nutritional experiments in unbiased preclinical animal models to assess the role of ω3-PUFAs in primary and secondary prevention of cardiovascular disease.

To study underlying genetic and epigenetic mechanisms of this polygenic disorder, genetically defined null allelic murine strains, including the LDLR-deficient mutant (*ldlr*−/−), lacking the LDL-receptor for LDL uptake, and the ApoE- (*apoe*−/−) mutant, devoid of the remnant and VLDL-ligand for cholesterol clearance by the LDL and LRP (LDL-receptor related), have been generated by transgenic techniques [5]. These two strains rapidly develop extensive atherosclerosis throughout the entire aorta and are congenial models for nutrition studies implementing atherogenic HFHC (“Western”) diets [[5], [6], [7], [8], [9], [10], [11], [12], [13], [14], [15], [16], [17], [18]]**.**

In this study, we used the unbiased, genetically defined auxotrophic *fads2*−/− mouse mutant [6] in sustained dietary studies to bypass potential confounding factors and to elaborate molecular roles of ω3-and ω6-PUFA deficiency in the multifactorial pathogenesis of atherosclerosis. The molecular bases of several phenotypic features of the seemingly unrelated multi-variant phenotype of the *fads2*−/− mouse have been uncovered so far.

Loss of the Δ6-desaturase in the *fads2*−/− mutant leads to a) shutdown of the biosynthesis of PUFAs from their precursor EFAs, with the complete loss of these essential structural elements in the architecture of membrane lipid bilayers; b) *Δ6-desaturase (fads2)* gene inactivation activating an aberrant pathway of linoleic acid (18:2) metabolism, resulting in the synthesis of the uncommon eicosa-5Z,11Z,14Z-trienoic acid (20:3^5,11,14^) end product; 20:3^5,11,14^ systemically substitutes all PUFAs as single surrogates in the fatty acid profiles of phospholipid and sphingolipid classes; c) the conditioned loss of precursor ω6-AA in the eicosanoid synthesis prohibiting thromboxane A2 and prostacyclin synthesis, required for platelet aggregation and thrombus growth in primary hemostasis of the clotting cascade, and leading to prolonged bleeding time and loss of leukotriene LTB4 synthesis in stimulated macrophages [6]; d) the altered lipid bilayer architecture of Sertoli and germ cells of testes and granulosa cells of ovaries disrupting intercellular junction systems and causing male and female infertility [6,7].

We focused our study on the impact of suppressed biosynthesis of ω3-and ω6-PUFAs on the development of atherosclerosis in the *fads2*−/− mouse.

To obtain insight into the underlying molecular role in the prevention or development of atherosclerotic lesions, we compared cohorts of male and female C57BL/6N and *fads2*−/− mice by exposition to two feeding regimens, which were started after weaning and sustained during the lifetime: 1) a standard chow diet, which contained the essential fatty acids (EFA), the precursors in the PUFA synthesis control C57BL/6N mice, and 2) a PUFA-free but EFA-containing HFHC (“Western”) diet. The results of these studies, which combine the unbiased genetic model and stringent feeding conditions, unveiled molecular links between the suppression of ω3-and ω6-PUFA synthesis and prolonged PUFA-free regular and high fat/high cholesterol diets, perturbed membrane lipidomes and lipid metabolism of liver, lipid droplets and lipoproteins. They led to the surprising observation that suppression of PUFA-synthesis in the *fads2−/−* mouse on neither a PUFA-free regular diet nor a prolonged PUFA-free HFHC (“Western”) diet triggered atherosclerotic plaque formation in the aortic arc, the thoracic and abdominal aorta.

The *fads2*−/− mutant proved resistant to the development of atherosclerotic lesions by sustained atherogenic diet.

Beyond this study, we pursued potential epistatic functions of the inactivated *fads2* locus and the PUFA deficiency in the development of atherosclerotic lesions in the *apoe*−/− and *ldlr*−/− mutants, two model systems frequently used in the study of the multifactorial molecular pathogenesis of atherosclerosis, which is characterized by dysregulated cholesterol and lipoprotein metabolisms and completely different from our system. We generated the *fads2−/− x apoe*−/− and *fads2−/− x ldlr−/−* double mutants for long-term feeding experiments with a regular chow and HFHC diet to straightforwardly demonstrate the impact of suppressed *fads2−/−* activity by comparing plaque formation to that in *apoe*−/− and *ldlr*−/− mutants. Suppression of PUFA synthesis in *fads2*−/− *x apoe*−/− and *fads2*−/− *x ldlr*−/− double knockout mice on normal chow or “Paigen” diet showed that neither enhanced nor reduced apoE and LDL-receptor deficiency mediated atherosclerotic plaque formation.

Immuno-histochemical observations strongly suggest the deleterious role of PUFAs as highly vulnerable chemical targets of autoxidation, releasing reactive aldehydes (e.g., HNE (4-hydroxynonenal)) and covalently modifying proteins to novel autoantigens, which trigger inflammatory responses in atherosclerotic lesions.

Our study demonstrates the necessity of a scrutinized view of the pleiotropic functions of ω3-and ω6-PUFAs as molecular culprits or benefactors during a lifespan before they are included in legitimate dietary recommendations.

## 2 Materials and methods

### 2.1 Mouse mutants, generation and genotyping

Generation and genotyping of *fads2+/−* and *fads2*−/− mice have been described before [6]. *Fads2+/−* mice were back-crossed 10 times into the *C57BL/N6* genetic background. *Fads2+/+* siblings of the heterozygous crossings were used as (+/+). *Apoe−/−* and *ldlr−/−* mice were crossed with *fads2+/−* mice to breed homozygous *fads2−/− x apoe−/− and fads2−/− x ldlr−/−* double mutants. Genotypes were led by PCR analysis of ear-punch DNA. The following oligonucleotide primers were used for genotyping of the *apoe*−/− and *apoe*−/− *x fads2*−/− double knockout loci: *apoe*−/−: 5′-GCC GCC CCG ACT GCA TCT -3′; *apoe*−/−: 5′- GCC TAG CCG AGG GAG GAC CG-3′; *ldlr*−/−: LDLR KO 5′- AATCCA TCT TGT TCA ATG GCC GAT-3′; LDLR COMMON: 5′- CCA TAT GCA TCC CCA GTC TT-3′; LDLR WT: 5′- GCA ATG GAT ACA CTC ACT GC-3′; *fads2−/−*: *5′-*UTRs 5′CCT TCC TTG TTC CAG ACA CGG TCT CAA GAG -3′and *fads2E1 3*′: *5*′*-* CGT AGC ATC TTC TCC CGA ATA GTG TCC GAT-3′.

Animals were housed in the specific pathogen-free (SPF) barrier mouse facility of the Center of Molecular Medicine (CMMC) with a 12h light/dark cycle and free access to water and chow. Cohorts of gender- and weight-matched and mutant mice were used in this study. The animal studies followed ARRIVE Guidelines [8]. Animal breeding and test protocols followed the principles and practices outlined in the Guide for the Care and Use of Laboratory Animals. The procedures were approved by the Institutional Animal Care and Use Committee of the University of Cologne and by the Landesamt für Natur, Umwelt und Verbraucherschutz Nordrhein-Westfalen.

### 2.2 Feeding regimens

The standard chow diet (Altromin diet #1310, Altromin, Lage, Germany) contained the two EFAs, linoleic acid (18:2) and α-linolenic acid (α-18:3), in all feeding regimens to prohibit EFA deficiency. Colonies of *+/+*, *fads2+/−* and *fads2−/−* mice were maintained on a regular (nd) diet (Altromin, Dinslage Germany), and two semi-synthetic high fat/high cholesterol diets (HFHC), alternatively named Western diets (Wd), were used to induce atherosclerosis: A) HFHC Western diet – 0.21% cholesterol, ssniff - Spezialdiäten GmbH, D-59494 Soest, approved to induce hypercholesterolemia, hyperlipidemia and atherosclerotic plaque formation in *apoe−/−* and *ldlr*−/− mice, and B) sodium cholate–supplemented HFHC diet (“Paigen” diet) [9].

Standardized feeding regimens of these diets were applied to cohorts of newborn female and male *fads2*−/− mice, starting after weaning at p21 and continuing for 4 months.

### 2.3 Cell fractionation of liver, isolation of lipid droplets and serum lipoproteins

Microsomal fraction of the 0.25M sucrose homogenate of liver was isolated as the 100,000×*g* sediment of the 12,000×*g* supernatant. Liver lipid droplets (LDs) were isolated from liver by established density gradient centrifugation adapted to liver homogenates [10]. In brief: mouse liver was homogenized in 2 mL disruption buffer (25 mM Tris–HCl, pH 7.4; 100 mM KCl, 5 mM EGTA, 1 mM EDTA) and diluted with 2 mL 1.098M sucrose in disruption buffer. The homogenate was spun for 10 min at 1500×*g* at 4 °C. The post-nuclear supernatant fraction was overlaid with 2.5 mL 0.27M and 0.13M sucrose and filled with top solution (25 mM Tris–HCl, pH 7.4, 1 mM EGTA and 1 mM EDTA) in the 11 mL centrifuge tube and spun at 36,000 rpm in the Beckman SW41 Ti rotor at 4 °C for 2 h. The top buoyant LD fraction was removed with a bent Pasteur pipette and suspended in lysis buffer, total volume 1 mL, and 10 more 1 mL fractions were isolated.

Serum lipoproteins were separated by agarose electrophoresis and cholesterol concentrations, determined using the Sebia HYDRASYS agarose gel electrophoresis system (Sebia, Inc. Norcross, Georgia, 30093, USA).

### 2.4 Laboratory measurements

Concentrations of blood glucose, serum insulin, triglycerides and total cholesterol of mice fasted overnight were determined by standard colorimetric assays [11].

### 2.5 Lipidome analysis

Total lipids from liver, subcellular fractions, LDs and serum of cohorts (*n* = 5) of adult (4-month-old) +/+ and *fads2−/−* female and male mice were extracted by homogenization in an Ultraturrax in 10 volumes of chloroform/methanol (CHCl_3_/CH_3_OH) at 2:1 (v/v) and re-extracted with CHCl_3_/CH_3_OH at 1:1 (v/v) and CHCl_3_/CH_3_OH at 1:2 (v/v) for 1 h each at 37 °C under a stream of nitrogen. The combined extracts of the total lipids were dissolved in CHCl_3_/CH_3_OH at 2:1 (v/v), washed with 2 M KCl and water and dried in a stream of nitrogen for separation of phospholipids by HPTLC (solvent system: methylacetate:1-propanol:chloroform:methanol:0.25% KCl 25:25:25:10:9 (v/v/v/v/v) or chloroform:ethanol:triethyl amine:water 60:70:70:14 (v/v/v/v)) and of neutral lipids (solvent system: hexane:ethyl ether:acetic acid 90:25:1 (v/v/v)) using HPTLC plates (Merck, Germany). Charred bands of phospholipids (PE, PI, PS, PC, SM, lyso-PC) and neutral lipid classes (C, TG, CE) were quantitated by densitometry using the ImageJ2 program (RRID: SCR_003070). The following representative PL and NL markers (Avanti Polar Lipids, Sigma) were used for semi-quantitative densitometry: 18:0-SM (MW 730), 18:0/18:2-PC (MW 786), 18:0/18:2-PS (MW 810), 18:0/20:4- PI (MW 904), 18:0/18:2-PE (MW 744), tri-oleoyl-glycerol (MW 885), 1,2-di-oleoyl-glycerol (MW 621), cholesterol (MW 386) and cholesteryl-oleate (MW 649).

For characterization of the diacylglycerol (DAG) core structure and quantitation of species of PL-classes by high-resolution full-scan MS/MS, PL classes were separated by HPTLC, as described above, visualized by Primuline fluorescence (0.2% in 80% acetone), collected on fritted glass filters, eluted with CHCl_3_/CH_3_OH at 2:1 (v/v) into Sovirel tubes and concentrated under N_2_. An Agilent Technologies (Waldbronn, Germany) HPLC system, coupled to an AB Sciex (Darmstadt, Germany) Triple TOF 5600 mass spectrometer, was used. The system was equipped with a Thermo Fisher Scientific (Dreieich, Germany) Accucore C8 (2.6 μm particle size, 50 × 3 mm) analytical column, and mobile phases consisted of aqueous formic acid 0.2% (pH 2) (solvent A) and acetonitrile as organic modifier (solvent B) for optimal ESI conditions. Thereby, the gradient was held for 0.5 min and then decreased from 50% A to 0% A with 0.325 mL/min within 6 min. The column was regenerated by washing for 1 min and equilibrated with 100% B for 3 min. The mass spectrometer was calibrated frequently (after 10 injections) via the Duo Turbo-V-Ion source by a calibrant delivery system containing the manufacturer's probes for positive ionization. The nitrogen for the ion source as well as collision gas supply was delivered by the nitrogen generator (CMC, Eschborn, Germany). Product ion experiments were acquired by isolating the respective [M+H]^+^ precursor ions in the quadrupole (unit resolution) and performing collision-induced fragmentation in the collision cell.

### 2.6 GC/MS analysis of fatty acid profiles of PL and NL classes

Total lipid extracts and isolated phospho- and neutral lipids were trans-esterified with 5% HCl-methanol at 80 °C for 1 h. One volume of water was added, and fatty acid methyl esters (FAMEs) were extracted with hexane and concentrated under nitrogen. When indicated, double bond positions were determined in DMOX-derivatized fatty acids as described before [11]. FAMEs were separated, identified and quantified by combined gas–liquid chromatography/electrospray ionization mass spectrometry (ESI-MS) on an Agilent (Waldbronn, Germany) 6890/5973N instrument equipped with an HP-5MS fused silica column (length 17 m, i.d. 0.25 mm, film thickness 0.25 μm) or on a Carlo Erba Instrument Model GC8000. Samples of FAMEs and 2,2-dimethyloxazoline derivatives were injected in a 10:1 split mode into the mass spectrometer, which was operated on full scan mode over a mass range of 50–500 u, and electron ionization (EI) was utilized at 70 eV. The injector temperature was set to 300 °C.

### 2.7 Protein analysis

Freshly dissected liver or subcellular fractions, aliquots of the LD fraction and serum of +/+ and *fads2*−/− mice were mechanically dispersed in lysate buffer containing protease inhibitor cocktail (Complete; Roche). Protein concentrations were measured using a Pierce BCA protein assay kit (Thermo Scientific).

Protein aliquots (100 μg) were separated by NuPAGE 4–12% Bis-Tris gels and transferred to nitrocellulose or PVDF (polyvinylidene fluoride) membranes using the NuPAGE Western blot system (Invitrogen). Blots were incubated overnight at 4 °C with respective primary antibodies and anti-GAPDH from Sigma–Aldrich (Cat# SAB5600208, clone RM114), anti-Caveolin from Santa Cruz Biotechnology (Cat# sc-7875, RRID: AB_2072020) and anti-Calnexin from Santa Cruz Biotechnology (Cat# sc-11397, RRID: AB_2243890) as internal standards. After washing and hybridization with conjugated secondary antibodies, blots were developed with the ECL system. Signals were quantified by densitometry using the ImageJ2 program (RRID: SCR_003070).

### 2.8 Gene expression analysis by qRT-PCR

RNA was isolated from control and *fads2−/−* liver using Trizol, Invitrogen. 10 μg of total RNA was reverse transcribed using a Transcriptase kit (Life Technologies, Darmstadt, Germany). Primer pairs used in quantitative PCR-reactions are listed in Hgprt was used as internal standard. qRT-PCR reactions were performed with the ABI Prism 7900HT, employing a 96-well format and the Fast SYBR Green Master Mix, Applied Biosystems, following the manufacturer's protocol. Data analysis was performed using the 2-ΔΔCt method.

### 2.9 Histology and immunohistochemistry

Mice were perfused from the left ventricle with cold PBS, then with PBS and PBS-buffered 4% paraformaldehyde. Tissues were fixed and processed for light- and immunofluorescence microscopy as described before [12].

For the assessment of atherosclerotic plaques, the entire freshly prepared aorta was isolated from the arch to the iliac bifurcation, adventitia and adipose tissue were thoroughly removed and the aorta was opened lengthwise, pinned flat on a mossy rubber pad and fixed in 10% neutral buffered formalin overnight. The entire aorta was stained for 10 min with oil red and *en face* preparations were digitally photographed and quantified using the Zeiss Imager M1 with the Axiovision software. Cryo- and paraffin-embedded sections (3 μm) of liver and rolled aorta were stained for lipid deposits with oil red. Aortic plaque components were immunohistochemically characterized using anti-Apo E from Acris Antibodies GmbH (Cat# BP2046, RRID:AB_972972), anti-Apo B 48/100 from Meridian Life Science (Cat# K23300R, RRID:AB_150526), anti-HNE from Abcam (Cat# ab46544, RRID:AB_722493), antibodies, for inflammatory parameters, anti-TNFα from Abcam (Cat# ab9739, RRID:AB_308774), anti-TNFαR from Santa Cruz Biotechnology (Cat# sc-1069, RRID:AB_632522), anti-NFκB p50 (Santa Cruz Biotechnology Cat# sc-114, RRID:AB_632034) and lysosomal markers anti-LAMP1 from Abcam (Cat# ab24170, RRID:AB_775978) and anti-SMPD1 [13] anti-serum ASMase. Lipid droplet markers: anti-Perilipin1 from Cell Signaling Technology (Cat# 9349, RRID: AB10829911), anti-rab5 from Abcam (Cat# ab109534, RRID: AB_10865740), anti-Tip47 (#GP37), anti-Perilipin2 (Cat# GP46), anti-DGAT-1 from Santa Cruz Biotechnology (Cat# sc-271934, RRID: AB_10649947) and anti-ATGL from Cell Signaling Technology (Cat# 2138, RRID: AB_2167955). A Zeiss microscope, Axio Imager M1 combined with the AxioVision imaging software (RRID: SCR_002677), a Slide Scanner Leica SCN400 with the Aperio ImageScope software (RRID: SCR_014311) and the inverted Leica TCS-SP laser scanning microscope with 63x objective were used.

### 2.10 Statistical analysis

Results are expressed as mean ± SEM. Statistical significance of differences between individual experimental groups was calculated by the unpaired *t*-test, using Graph Pad Quick Calcs: t-test calculator. p-values of ≤0.05 ∗, ≤0.01 ∗∗, ≤0.001 ∗∗∗ were considered significant.

## 3 Results

We investigated through long-term dietary feeding experiments in cohorts of +/+ and *fads2−/−* mice a) the role of PUFA-free regular chow and b) PUFA-free HFHC diet on molecular parameters of hepatic lipid metabolism, c) how the absence of ω3-and ω6- PUFAs modifies the dynamics of lipid droplet structures and d) the serum lipoprotein pattern and e) development of atherosclerotic lesions in the aorta.

### 3.1 Kinetics of the deprivation of the lipidome of newborn *fads2*−/− mice from ω3-and ω6-PUFA

*Fads2*−/− male and female mice are infertile. Breeding of *fads2*+/− or of *fads2*−/− male and female mice, raised on sustained AA/DHA-supplemented diet that started after weaning, yielded the required *fads2−/−* colonies. Analysis of the lipidome of newborn *fads2−/−* mice and of +/− and +/+ foster mothers revealed identical ω3-and ω6-PUFA patterns.

**Figure 1:**
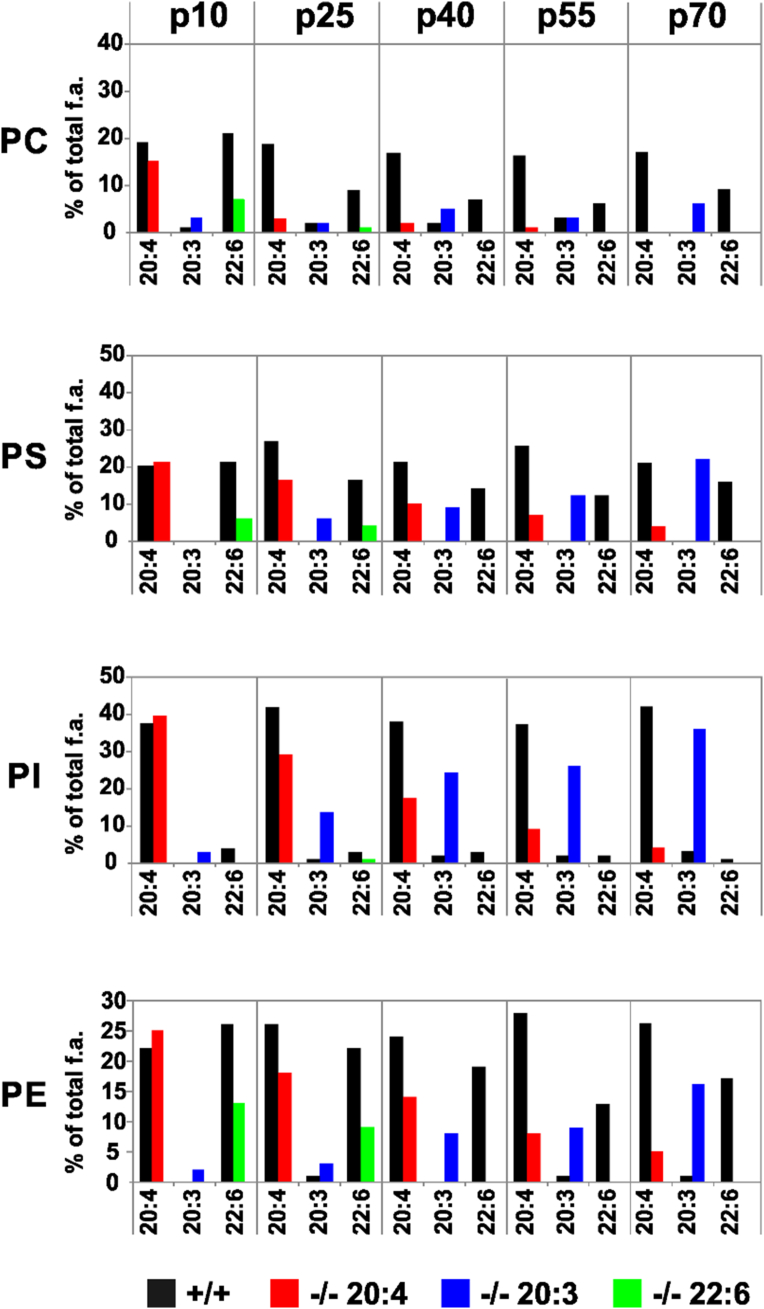
Specific modification of the PUFA pattern of phospholipidomes of liver *of fads2−/−* newborn by PUFA-free diet during five intervals within 70days after birth. Only key representatives ω3-22:6 and ω6-20:4 of the PUFA pattern and the surrogate 20:3^5,11,14^ acid have been selected from the complete analysis presented in SI Figure 2.

We therefore followed the kinetics of the modification of the fatty acyl pattern of the main PL-classes of liver in cohorts of control and *fads2−/−* newborn mice, receiving the regular PUFA-free control chow over a period of 70 days. The regular chow contained only the essential fatty acids ω6-linoleic and ω3-α-linolenic acid. ω6-AA and ω3-DHA were used as representatives of the ω3-and ω6-PUFA families in the bar diagram of the regression analysis (Figure 1). The complete analysis of all PL classes of liver of control and *fads2*−/− mice is presented in SI Figure 2**.**

DHA was depleted from all PL classes at p30, and the linear increase of 20:3^5,11,14^ reached the concentration of AA of the respective PL class, particularly in PE, PS and PI of liver of the developing *fads2*−/− mice (Figure 1). Unlike ω3-DHA, ω6-AA −/− was replaced slower by the surrogate 20:3^5,11,14^. Trace concentrations of AA in PI, PE and PS persisted at p70.

We then elaborated the relevance of the inactivated *fads2−/−* locus in the genome of the *fads2*−/− x *apoe*−/− double mutant for the development and progression of atherosclerotic lesions in the aorta. Cohorts of age-matched male and female control *C5*7BL*/6n, fads2*−/−*, apoe*−/− and *fads2*−/− x *apoe*−/− mice were subjected to a) regular chow and b) the HFHC diet for a period of 4 months. c) To enhance the formation and development of aortic lesions in *apoe*−/− and *ldlr*−/− mutants, a HFHC diet supplemented with 0.5% sodium cholate and 1% cholesterol (“Paigen” diet) was administered for a period of eight months after weaning.

### 3.2 Impact of ω3-and ω6-PUFA–free regular and HFHC (“Western”) diet on liver lipid metabolism

Cohorts of *+/+, fads2*−/−*, apoe*−/−*, fads2*−/− x *apoe*−/− and *fads2*−/− x *ldlr*−/− were studied in ω3-and ω6-PUFA–free regular and HFHC (“Western”) diets for a period of four months after weaning. All animals survived the sustained, long-term chow-fed and HFHC-fed dietary interventions.

Body weight of male and female cohorts, independent of the genotype, differed by approximately 25%. At the end of the feeding period the body weight of +/+ mice exceeded that of the mutant strains by 20%. (SI Figure 1A-D).

Carbohydrate metabolism of +/+ *and fads2*−/− on sustained regular diet (Figure SI 1E) and male and female *+/+, fads2−/−*, *apoe−/−* and *fads2−/−* x *apoe−/−* mice on the HFHC diet at p120 **(**Figure SI G**)** remained unaffected by the absence of PUFAs, as indicated by unaltered fasting blood glucose concentration, and serum insulin concentration of *+/+* and *fads2*−/− mice on regular chow **(**Figure SI 1F).

**Figure 2:**
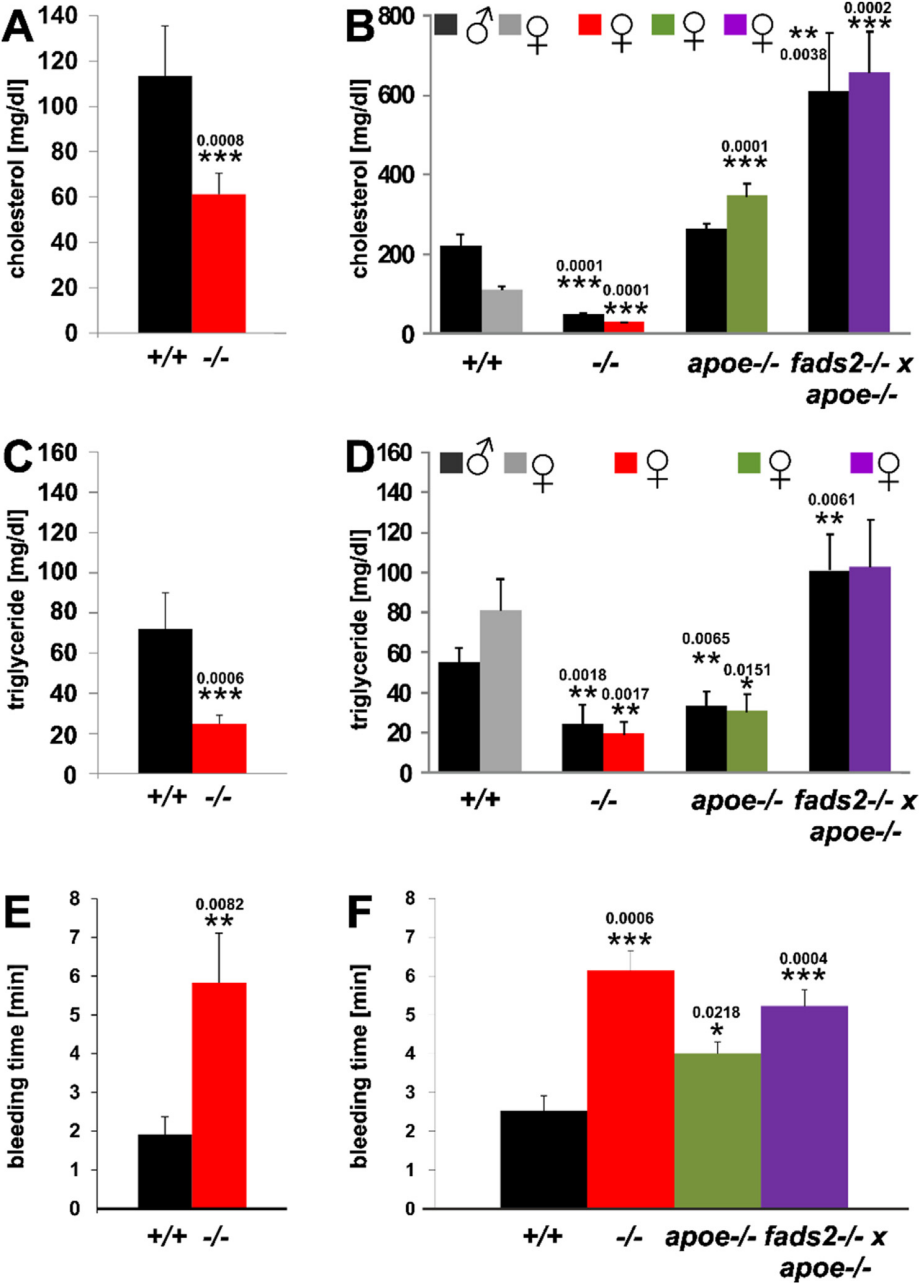
PUFA deficiency causes hypocholesterolemia and hypotriglyceridemia in *fads2−/−* male and female mice on nd (normal) and HFHC diets. Serum concentration of cholesterol in (A) and of serum triglycerides in (C) of +/+ and *fads2−/−* mice on normal chow. Serum concentration of cholesterol (B) and of triglycerides (D) in male and female *+/+, fads2−/−* and *apoe−/− and fads2−/− x apoe−/−* mice on HFHC diet. (E) Bleeding times of +/+ and *fads2*−/− mice on normal chow, (F) *+/+, fads2*−/− and *apoe*−/− and *fads2*−/− *x apoe*−/− mice on HFHC-diet. Size of cohorts in (A), (C) and (E) *n* = 20, in (B), (D) and (F) *n* = 5.

Analysis of total serum cholesterol and triglycerides of *fads2−/−* mice on nd (normal diet) revealed hypocholesterolemia and hypotriglyceridemia: C and TG concentration were reduced to half of those of +/+ control mice (Figure 2A,C). The HFHC diet increased serum cholesterol to twice the concentration of +/+, but did not change the low C concentration in cohorts of male and female *fads2−/−* mice (Figure 2B,D). Inactivation of the *fads2* locus in male and female *fads2−/−* x *apoe−/−* mice elevated serum C and TG concentrations to two- and threefold those in *apoe−/−* mice, respectively.

Our previous study has demonstrated the inactivation of the clotting system by the deprivation of arachidonic acid (20:4), the precursor of antithrombotic thromboxane A2 synthesis, leading to prolonged bleeding time, another hallmark of the *fads2*−/− phenotype [6]. Serum concentration of TXB2, the stable metabolite of TXA2, was not measurable by the immune-assay in the *fads2−/−* serum. Average bleeding times of *nd-fads2*−/− was 6–8 min, compared to 2–2.5 min in +/+ mice (Figure 2E). HFHC diet had no impact on the prolonged bleeding time of the *fads2*−/− and *fads2*−/− x *apoe*−/− mutants (Figure 2F).

**Figure 3:**
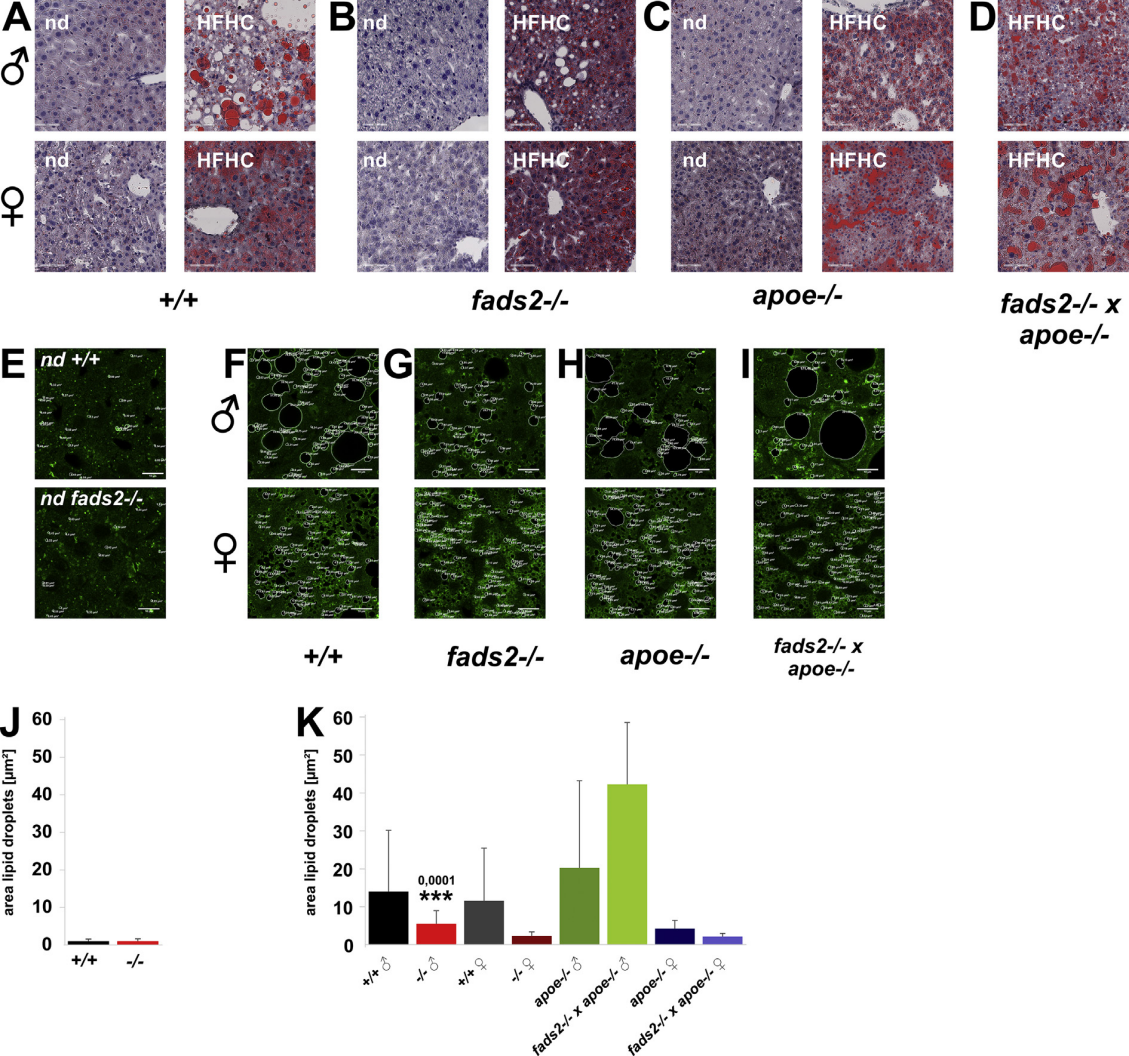
PUFA deficiency modifies liver lipid metabolism, LD formation and micro-droplet steatosis in male and female *fads2*−/−mice during a 120-d feeding period of regular chow and HFHF-diet. Oil red-stained sections of liver of males and females on normal chow (nd) and on HFHC diet (A) +/+, (B) *fads2*−/−, (C) *apoe*−/− and (D) *fads2*−/− x *apoe*−/− mice. Bar diagram of average LD sizes of normal chow fed (J) +/+ and *fads2*−/− and HFHC-fed (K) male and female *+/+*, *fads2*−/−*, apoe*−/− and *fads2*−/− *x apoe/-* mice. Planimetry of LD's of male and female on normal chow (E) and HFHC diet (F) +/+, (G) *fads2*−/−, (H) *apoe*−/− and (I) *fads2*−/− x *apoe*−/− mice. *n* = 100 LDs.

Microscopic images of sections of liver of male and female *+/+, fads2−/−, apoe−/−* and *fads2−/−* x *apoe−/−* mice maintained on the regular control and the HFHC diet were stained with oil red for quantitation of lipid deposition in lipid droplets (LD) (Figure 3A–D). Livers of the four different genotypes revealed different degrees of hepato-steatosis. We improved this quantification by immune-staining lipid droplets in paraffin sections of liver using the anti-perilipin2 antibody that recognizes LD-specific perilipin2 integrated into the LD membrane (Figure 3E–I). The sharp demarcation provided a detailed view of size, number, intra- and extracellular localization and LD. Quantitation of LD number and size is summarized (Figure 3J,K).

### 3.3 Absence of ω3-and ω6-PUFAs perturbs membrane phospholipid bilayer of ER and of LD phospholipid monolayer of *fads2−/−* mouse liver

A molecular hallmark of the *fads2*−/− phenotype is the systemic substitution of ω3- and ω6-LC–PUFA by surrogate 20:3^5,11,14^ (sciadonic acid), synthesized from linoleic acid by an aberrant pathway [11].

We first investigated the impact of a sustained long-term regular chow dietary regimen on liver lipid metabolism of +/+ and *fads2*−/− cohorts. Comparative experiments focused on the molecular architecture of the membrane lipid bilayer of the ER (microsomes), the site of lipid synthesis, the assembly of serum lipoproteins, and the ER-derived lipid monolayer of lipid droplets, the intracellular energy storage particles of the liver.

**Figure 4:**
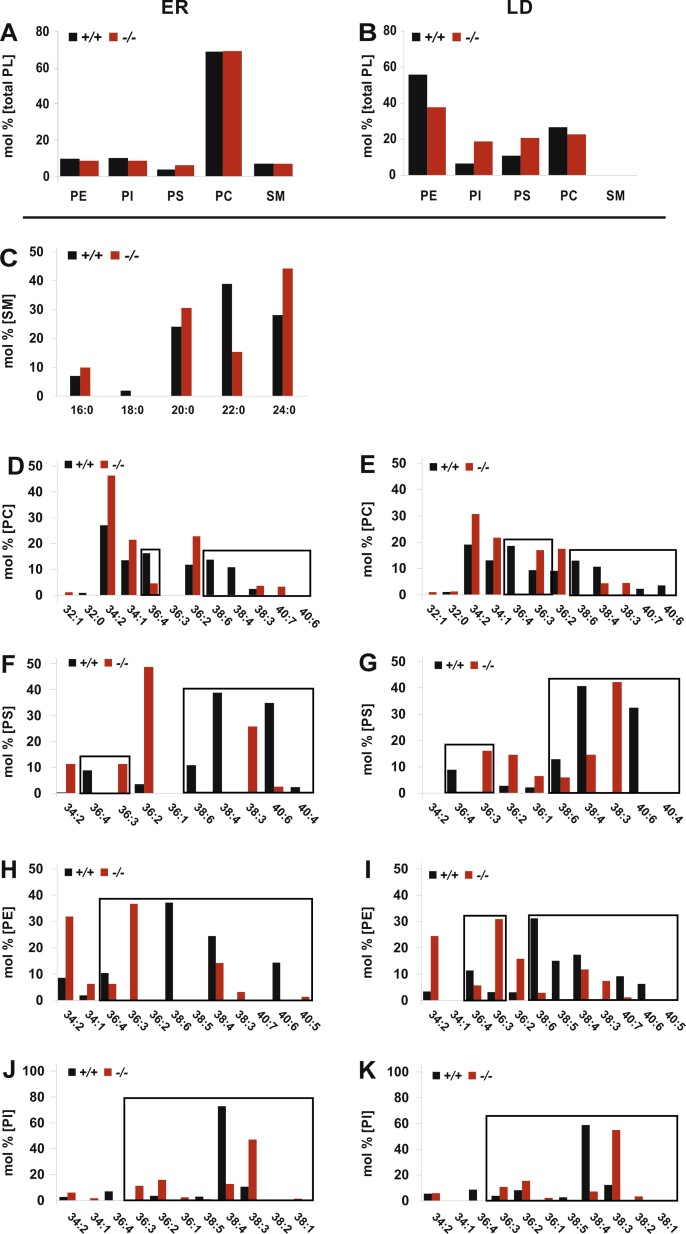
PUFA deficiency disrupts the hydrophobic DAG-core structures of PL-classes in the bilayer-lipidome of the ER and LD of +/+ and *fads2*−/− liver maintained on prolonged (4months) regular chow. Bar diagrams of densitometric quantitation of PL bands of total lipid extracts, separated by HPTLC. Mol% of total PL fraction (A) ER and (B) LD of +/+ and *fads2*−/− mice. ER: Mol% of (C) fatty acyl-substituents of ceramides of SM species, of (D) PC, (F) PS, (H) PE and (J) PI, and LD: (E) PC, (G) PS, (I) PE and (K) PI. PL classes were separated by HPTLC in solvent system: methyl acetate: 1-propanol: chloroform: methanol: 0.25% KCl 25:25:25:10:9 (v/v/v/v/v). Encasements highlight the modified DAG-species in individual PL-classes.

ER and LDs were isolated by established gradient ultra-centrifugation [10]. PL and NL classes of the ER membrane lipid bilayer and LD lipid monolayer were separated by HPTLC for MS/MS analysis of the profile and stoichiometry of the hydrophobic DAG core species of individual PL classes (Figure 4), and their fatty acyl substituents were further characterized by GC/MS. NL classes of ER were isolated by HPTLC for GC/MS analysis (SI Figure 3).

**Figure 5:**
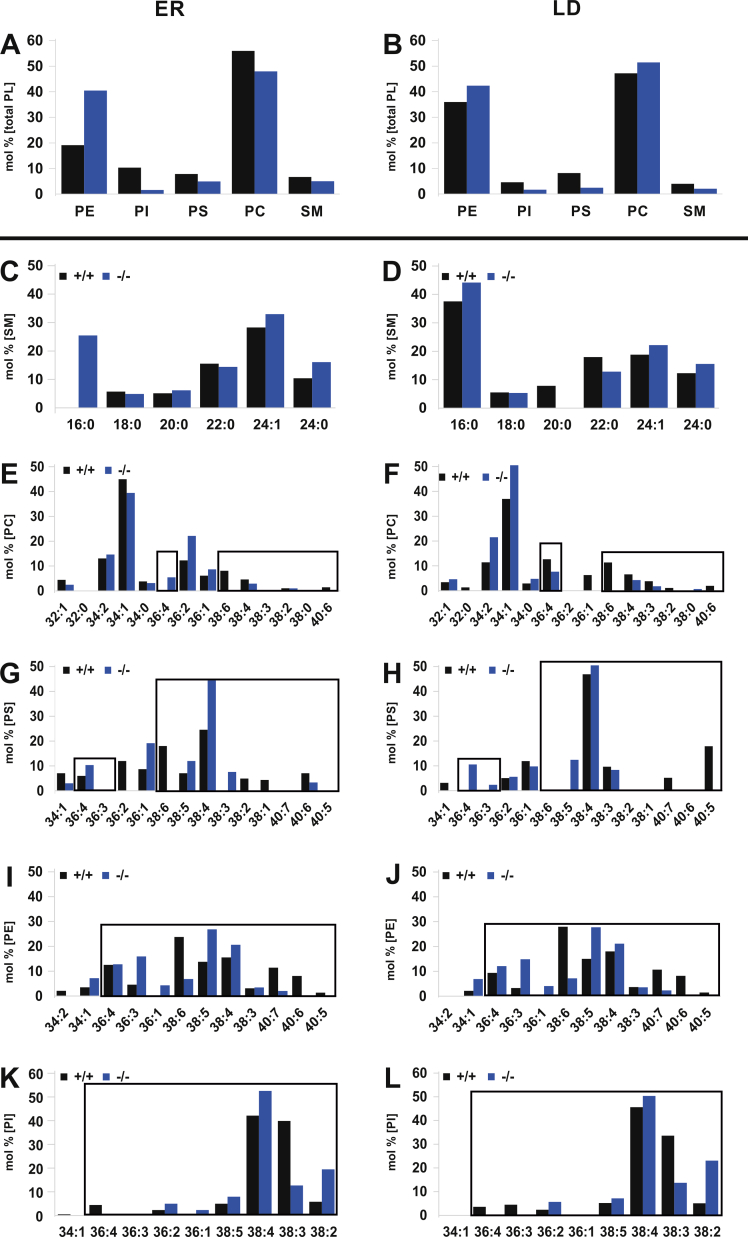
Sustained HFHC diet (4mo) suppresses 20:3 synthesis and PUFA substitution in the phospholipidome of the bilayer of ER and LD of +/+ and *fads2*−/− liver. Bar diagrams of densitometry of PL bands of total lipid extracts, separated by HPTLC. Mol% of total fraction of +/+ and *fads2*−/− (A) ER (B) LD. ER: (C) SM, (E) PC, (G) PS and (I) PE, (K) PI and +/+ and *fads2*−/− LD: (D) SM, (F) PC, (H) PS, (J) PE and (L) PI. PL classes were separated by HPTLC in solvent system: methyl acetate: 1-propanol: chloroform: methanol: 0.25% KCl (25:25:25:10:9) (v/v/v/v/v). Encasements highlight the modified DAG species in individual PL-classes.

The stoichiometry of PL classes was very similar in the lipidomes of the ER and LD of +/+ and *fads2*−/− liver (Figure 4A,B). However, MS/MS profiling of the DAG core structures of PL classes revealed profound structural modifications of the fatty acyl substituents of all PL classes of the entire ER and LD phospholipidomes.

Three major PS species, 38:6 (16:0/22:6), 38:4 (18:0/20:4) and 40:6 (18:0/22:6) in the ER and LD phospholipidomes of +/+ liver, contrasted those of the ER and LD of *fads2*−/− liver: 36:3 (16:0/20:3) 38:3 (18:0/20:3) and 38:4 (18:1/20:3) (Figure 4F–G).

The PE fraction of +/+ ER and LD consisted largely of the ω3-22:6 containing DAG species 38:6 (18:0/22:6), and 40:7 (18:1/22:6), 40:5 (18:0/22:5) and ω6-20:4 containing 38:4 (18:0/20:4) and 38:5 (18:1/20:4), whereas the PE fraction of the ER lipid bilayer and the LD lipid monolayer of the *fads2−/−* were enriched with linoleic acid (18:2) in the DAG species 34:2 (16:0/18:2) and with 20:3^5,11,14^ in the 36:3 (16:0/20:3), 36:4 (16:1/20:3) and 38:3 (18:0/20:3) species.

Only one dominant species, 38:5 (18:1/20:4), represented the PI fraction of the +/+ ER and LD membranes, which was replaced in the PI of *fads2−/−* ER and LD by 36:3^5,11,14^ (16:0/20:3), 36:4 (16:1/20:3), 38:3 (18:0/20:3) and 38:4 (18:1/20:3).

Unlike the lipidome of the ER membrane, the LD-phospholipidome of +/+ and *fads2−/−* liver was devoid of SM (Figure 4B). Absence of SM and accumulation of PS, PI and PE further indicate the outer cytoplasmic lipid leaflet of the asymmetric ER lipid bilayer as donor of the LD–PL monolayer.

**Figure 6:**
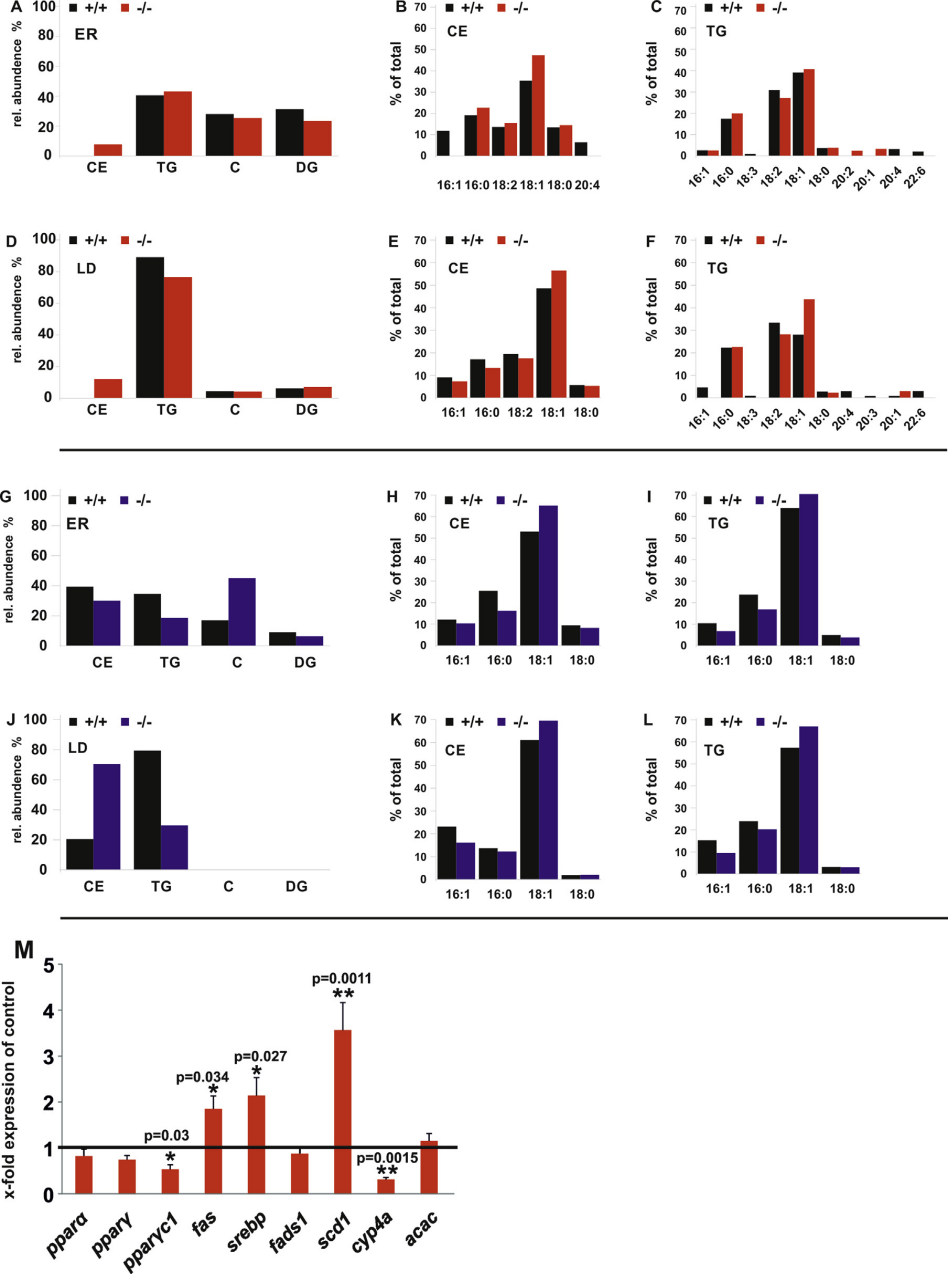
Comparison of NL classes and fatty acyl-profiles in the lipidome of ER and lipid core of LDs of liver of +/+ and *fads2*−/− mice on long term (4mo) regular chow. Bar diagrams of densitometry of NL bands of total lipid extracts. Relative abundance (mol%) of CE, TG, C, DG in total NL fraction of (A) ER, (D) LD of +/+ and *fads2*−/− mice on regular diet, and (G) ER, (J) LD of +/+ and *fads2*−/− mice on HFHC diet. HPTLC-separation in solvent system: hexane/ethyl ether/acetic acid 90/25/1 (v/v/v). GC/MS analysis of methylesters of fatty acid pattern of neutral lipids of LD of +/+ and *fads2*−/− mice on regular chow of ER (B) CE and (C) TG, and of LD (E) CE and (F) TG and on HFHC diet of ER (H) CE, (I) TG, of LD (K) CE and (L) TG. (M) Gene expression of key transcription factors and enzymes in liver of f*ads2*−/− mice relative to +/+ mice (black bar, expression = 1) on regular, PUFA-deficient chow.

Regular chow raised only the TG concentration in the NL profile of the globular hydrophobic core of LDs in *fads2−/−* liver. DG and CE were present in minor concentrations and C was not detectable. Stoichiometry of CE, TG, C and DG in the NL fractions of ER and LDs of +/+ and *fads2*−/− liver on regular diet were closely related, as were the fatty acid patterns of CE and TG. LDs of +/+ and *fads2−/−* liver of HFHC-fed mice contained only CE and TG, the CE concentration being increased fourfold and TG concentration reduced threefold in *fads2−/−* LDs (Figure 6J).

Gene expression of key transcription factors and enzymes in liver of +/+ and *fads2−/−* mice on regular, PUFA-deficient chow (Figure 6M) indicated elevated steady-state mRNA concentration of lipogenesis regulating srebp1c, fatty acid synthase fas and remarkable overexpression of stearoyl-CoA desaturase (scd1) and suppression of the P450 cytochrome hydroxylase (cyp4A) involved in the metabolism of PUFAs leading to physiologically important metabolites.

### 3.4 PUFA deficiency impairs lipid droplet size and fusion

**Figure 7:**
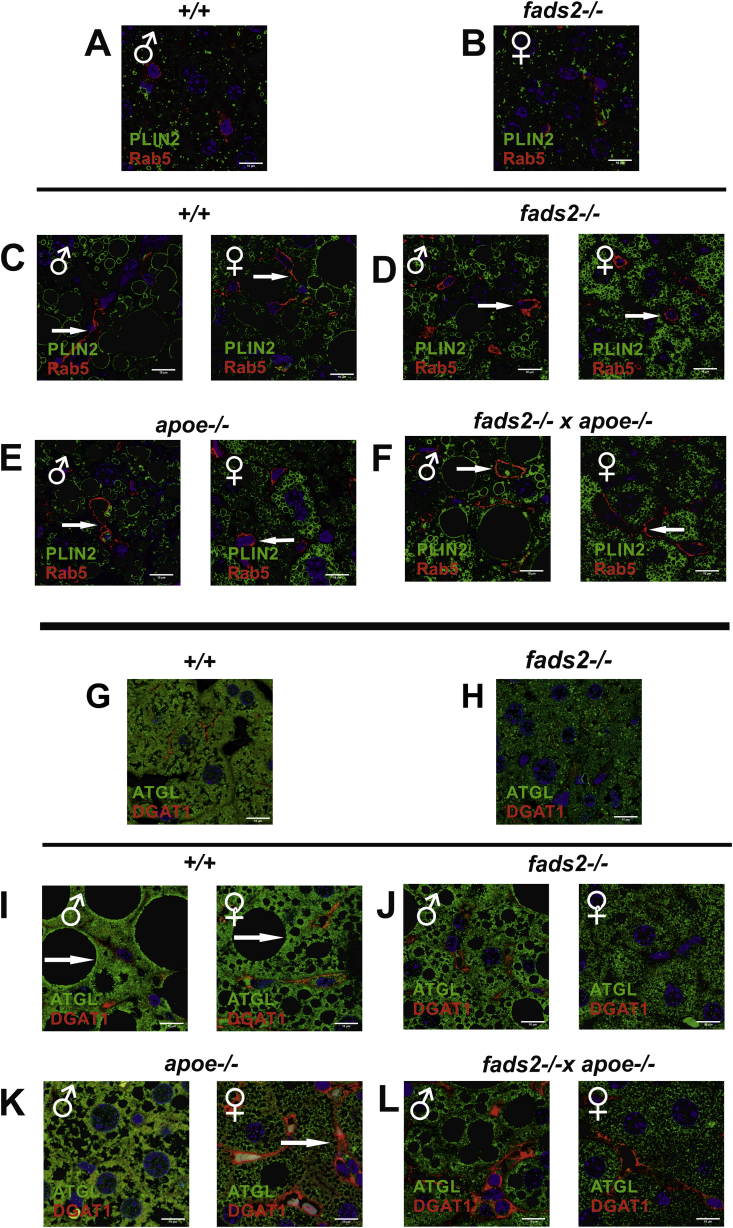
LD dynamics in *fads2−/−* liver of mice on regular chow and HFHC-diet. Merged IF-images of anti-PLIN2 and anti-Rab5 antibody stained liver sections of (A) +/+, (B) *fads2−/−* mice on regular diet, males and females on HFHC (C) +/+, (D) *fads2−/−,* (E) *apoe−/−,* (F) *fads2−/− x apoe−/−*. Merged IF-images of liver sections of males and females stained with LD-specific anti-ATGL and anti- DGAT1 antibodies. Regular diet (G) +/+, (H) *fads2−/−* and HFHC diet (I) +/+, (J) *fads2−/−,* (K) *apoe−/−* and (L) *fads2−/− x apoe−/−*.

**Figure 8:**
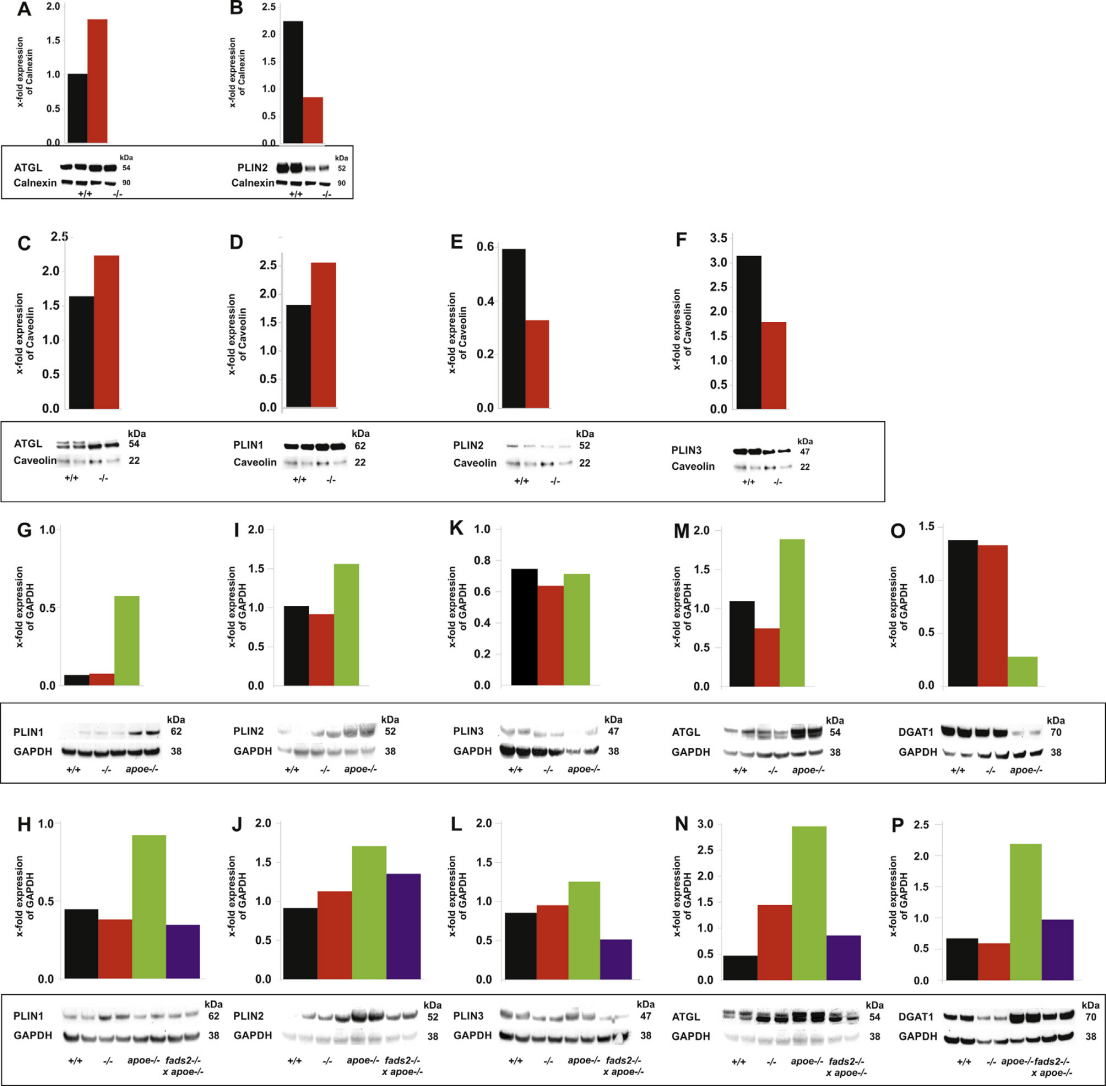
PUFA deficiency perturbs the regulation of enzymes of TG influx- and efflux-regulatory proteins of liver lipid droplets. Western blot analysis of the expression protein lysates using antibodies recognizing ATGL and PLIN2 (ADRP in (A,B) ER and (C–F) LDs of +/+ and *fads2−/−* mice on regular diet. Expression in liver of +/+, *fads2−/−* and *apoe−/−* mice on regular (nd) diet of (G) PLIN1, (I) PLIN2, (K) PLIN3 (M) ATGL, (O) DGAT1 and on HFHC diet, including the lysate of *fads2*−/− *x apoe*−/− liver: (H) PLIN1, (J) PLIN2, (L) PLIN3, (N) ATGL, (P) DGAT1.

We next expanded the lipidomic analyses by immuno-histochemical (Figure 7) and Western blot analysis of key proteins intimately involved in LD formation, uptake of fatty acids, and genes of enzymes regulating TG synthesis and lipolysis (Figure 8). Western blot analysis of lysates of liver and LDs included the dominant LD surface-bound perilipins, members of the PAT protein family, PLIN1 (Perilipin1), PLIN2 (Perilipin-2, ADRP, Adipophilin) and PLIN3 (TIP47, tail interacting protein), which, associated with ATGL (adipose triglyceride lipase) and DGAT-1 (diacylglycerol acyl transferase-1), regulates LD formation, growth and fusion LD formation (Figure 8) [14,15]. DGAT1 catalyzes the final step in TG synthesis and storage in hepatocytes and stellate cells, located between the antiluminal side of sinosoidal endothelial cells and the basolateral surface of hepatocytes in the space of Disse.

Sections of liver of PUFA-deficient male and female *fads2−/−* mice on regular chow (Figure 7A,B) were free of LDs and, in response to HFHC diet (Figure 7C,D), stored TG in micro-vesicular LD, indicated by LD-specific PLIN2 expression. We included small Rab5 GTPase, known as a key player in the regulation of LD multi-protein of membrane assembly, fusion and cytoskeletal transport [16]. Small GTPase Rab5 docking sites, distributed across the entire plasma membrane of control liver stellate cells and responsible for the fusion of small LD, were aggregated and LD fusion–inhibited in *fads2−/−* liver (Figure 7D). In addition, immunohistochemistry of liver sections of mice on the regular diet stained with anti-ATGL and anti-DGAT1 (Figure 7G-L) clearly visualized the LD membrane harboring ATGL and DGAT1 and indicated low expression of ATGL and DGAT1 similar to PLIN2 and Rab5 in merged images of the livers of control and male and female *fads2−/−* mice on regular chow, stained with ATGL and DGAT1 antibodies (Figure 7G,H). Male and some female *fads2*−/− mice on the HFHC diet had developed micro-lipid droplet steatosis: (I) *+/+*, (J) *fads2*−/−*.* Strong expression was observed in (K) *apoe*−/− mice and micro-vesicular LDs and attenuated expression of ATGL and DGAT1 in (L**)** *fads2*−/− x *apoe*/- mice.

The downregulation of PLIN2 (ADRP) and upregulation of ATGL expression in ER and LDs of *fads2−/−* mice were reflected in the decrease of LD-TG. Perilipin 1, located in the lipid droplet monolayer coat and known to serve as a recruitment site for lipases, blocks ATGL-catalyzed TG hydrolysis but triggers lipolysis upon phosphorylation by protein kinase A [17]. Western blot analysis (WB) of PAGE-separated protein lysates of +/+ and *fads2−/−* liver and LDs revealed upregulated PLIN1 expression in *fads2−/−* LDs. Suppression of PLIN3 (TIP47) in *fads2−/−* liver correlated with the intra-hepatocyte accumulation of LDs.

### 3.5 PUFA synthesis–deficient ER perturbs phospho- and neutral lipidomes of serum lipoproteins

**Figure 9:**
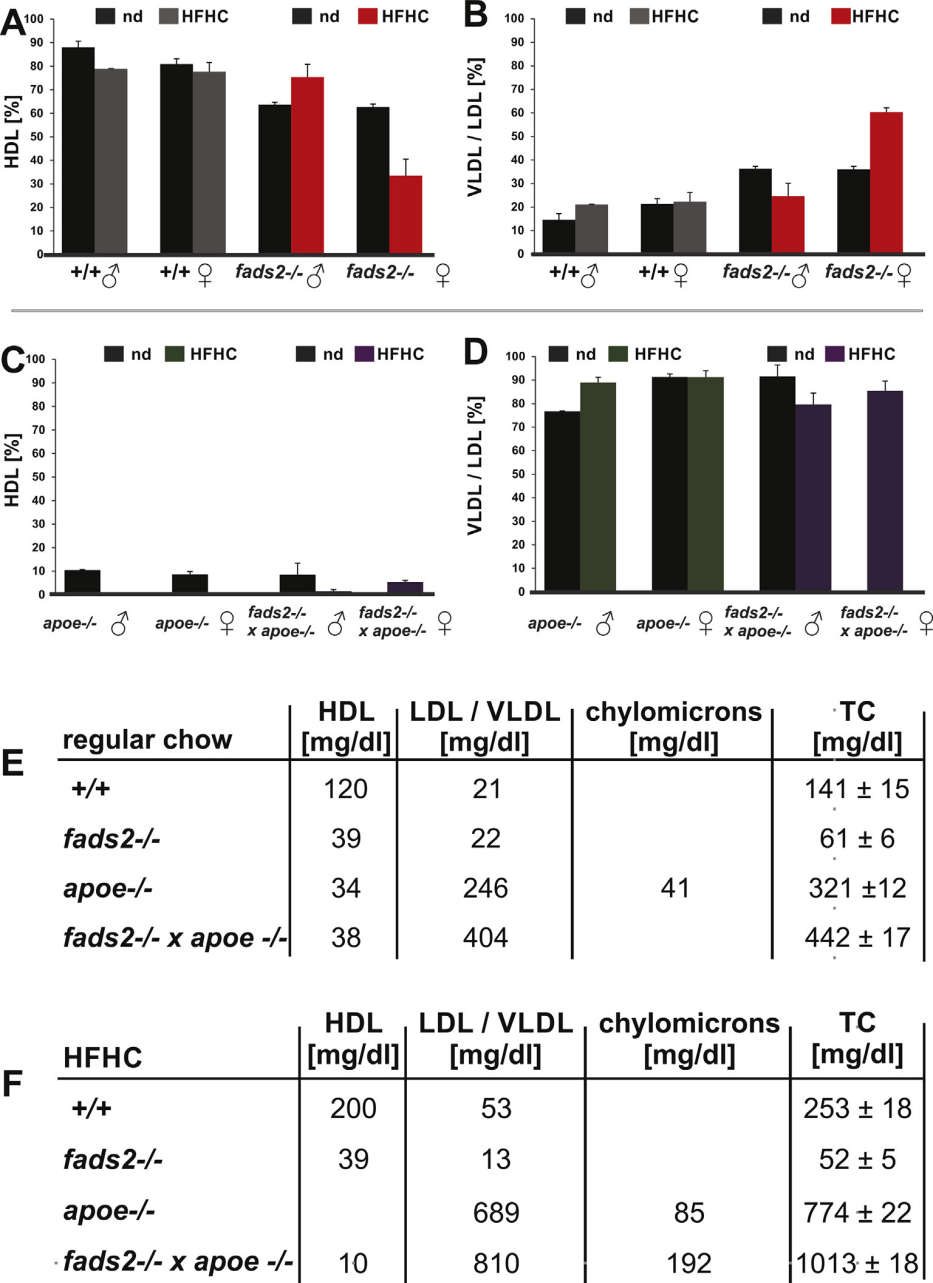
Serum-Lp pattern of male and female control and *fads2−/−* mice on regular and HFHC diet separated by agarose gel electrophoresis. Relative concentration of serum-LP fractions of adult (4mo) male and female +/+, *fads2−/−, apoe−/−* and *fads2−/− x apoe−/−* mice on sustained normal (nd) and HFHC-diet. (A) HDL concentration, (B) VLDL/LDL concentration of +/+ and *fads2−/−,* (C) HDL concentration *apoe−/− and fads2−/− x apoe−/−* mice. (D*)* VLDL/LDL concentration of *apoe−/− and fads2*−/− *x apoe−/−* mice. Absolute cholesterol concentration in Lp fractions of *+/+*, *fads2−/−, apoe−/−* and *fads2−/− x apoe−/−* male mice on (E) regular chow and (F) HFHC diet.

Serum Lp-profiles of adult (4-month-old) male and female *+/+, fads2−/−, apoe−/−* and *fads2−/−* x *apoe−/−* mice on long-term normal and HFHC diets were established by Sebia HYDRASYS agarose gel electrophoresis system (Figure 9, SI Figure 4). Under regular chow, HDL and VLDL/LDL serum lipoproteins of +/+ and *fads2−/−* male and female mice remained unchanged. The exception was female *fads2−/−*, which showed reduced HDL but elevated VLDL/LDL concentrations (Figure 9A,B). HFHC diet strongly suppressed HDL in the serum of the *apoe−/−* and *fads2−/−* x *apoe*−/− double mutant (Figure 9C).

Suppression of ω3-and ω6-PUFA synthesis elevated VLDL/LDL in female *fads2*−/− mice, but did not reduce the concentration of VLDL/LDL in male and female *fads2−/−* x *apoe−/−* mutants (Figure 9D). Quantification of total serum cholesterol (TC) and of C in HDL, VLDL, LDL and chylomicrons of control and *fads2−/−, apoe−/−* and *fads2−/−* x *apoe−/−* mice on regular and HFHC diet is summarized in (Figure 9E,F).

**Figure 10:**
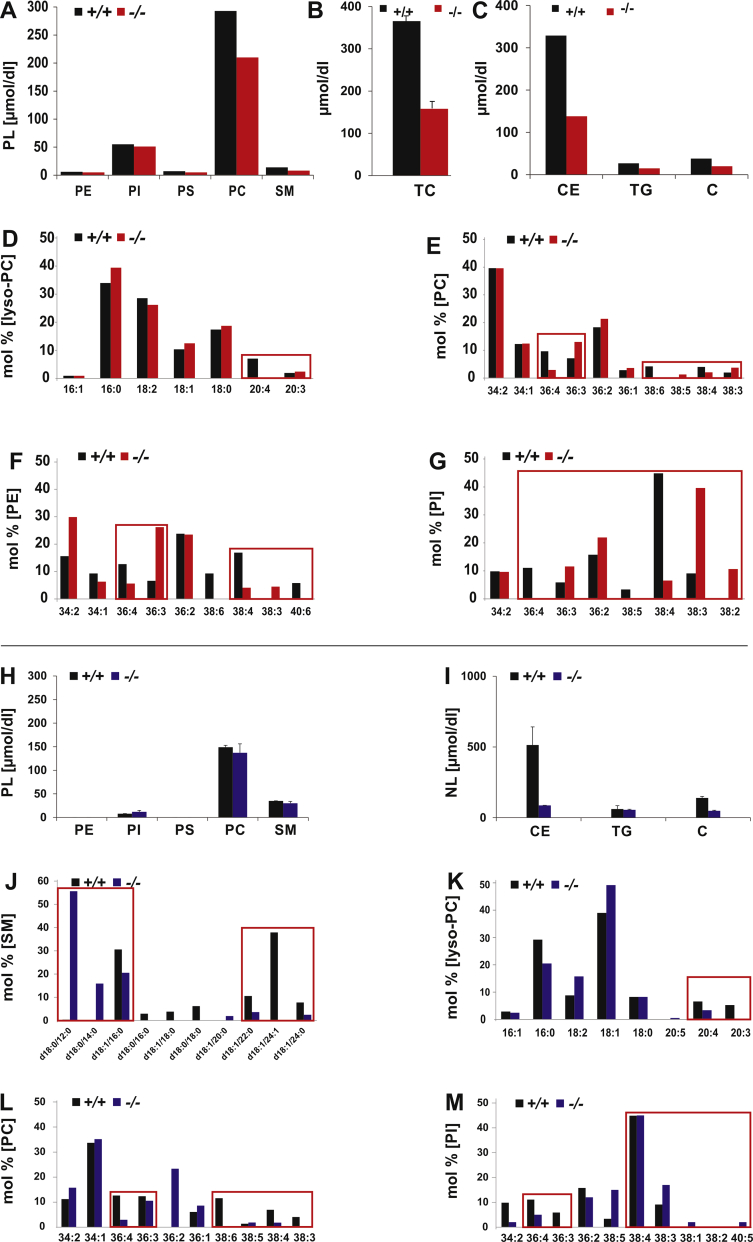
PUFA deficiency perturbs the hydrophobic DAG core structures of PL and NL classes of serum lipoproteins of control and *fads2*−/− mice on regular diet. Bar diagrams of profiles of concentration of (A) PL (B) total cholesterol (TC) and (C) NL classes. Mol % of total fatty acyl-residues of PL-species of (D) lyso-PC, (E) PC, (F) PE and (G) PI. Concentration of: (H) PL and, (I) total cholesterol, (J) NL classes of serum lipoproteins of *fads2*−/− mice on HFHC diet. Mol% of total fraction of (K) Ceramide species of SM, fatty acyl residues of (L) lyso-PC of DAG species of (M) PC, (N) PI. Encasements highlight the altered Ceramide in SM and DAG-profiles in PL classes.

The steady-state profile of the PL classes of total serum lipids in regular diet–fed +/+ and *fads2*−/− mice contained PC as main and PI as minor PL class, which remained rather stable during the feeding period, unlike that of NL lipidome, in which concentrations of C, CE and TG in serum of *fads2−/−* mice were attenuated to a low concentration (Figure 10C). HFHC diet decreased the PC/SM ratio and PI to the abundance of 5 mol% in the phospholipidome; the low C, CE and TG concentrations in the NL pattern remained stable.

Species analysis by MS/MS uncovered SM species in *fads2−/−* Lp highly substituted with C12, C14 and C16 in addition to the prevailing 16:0 and 24:1-substituted SM species (Figure 10K). DAG species of the major PL classes, PC, PE and PI, are characterized by the exchange of 20:4 with 20:3 in PC and PI in *fads2−/−* serum Lp, highlighted by the encasements (Figure 10D–G).

HFHC-diet left the profile of PL-classes of +/+ and *fads2*−/− lipoproteins unimpaired (Figure 10H). The DAG core structures of PL species, however, revealed profound structural changes. HFHC diet strongly suppressed 20:3^5,11,14^ synthesis, which otherwise substituted all ω3-and ω6- PUFAs in lyso-PC, PC, PI and PE.

**Figure 11:**
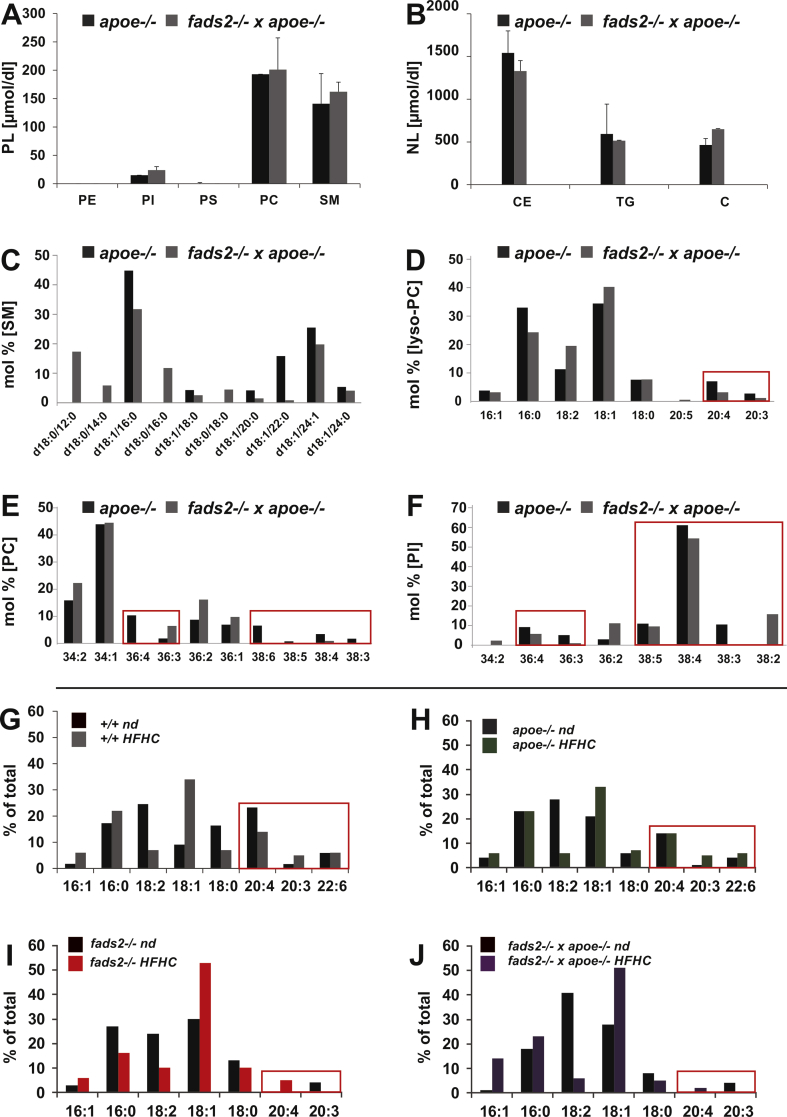
PUFA deficiency perturbs the hydrophobic DAG-core structures of PL and NL classes of serum lipoproteins of *apoe*−/− and *fads2*−/− x *apoe*−/− mice on HFHC diet diet. Bar diagrams of profiles of concentration of (A) PL (B) total cholesterol (TC) and (C) NL classes. Mol % of total fatty acyl residues of (D) ceramides of SM-species, (E) lyso-PC, (F) PC, (G) PI. Steady state fatty acid profiles of total lipids of serum Lps of H) +/+, **(**J) fads2−/−, (I) *apoe*−/−*,* (J*) fads2*−/− *and (K) fads2*−/− *x apoe*−/− mice on regular and HFHC diet. Encasements highlight the severely altered DAG profiles in PL classes. Values are average of three analytic replicates of pooled sera of four age- and gender-matched mice of each genotype.

The two dominant classes in the serum PL profiles of the *apoe−/−* and *fads2−/−* x *apoe−/−* cohorts are PC and SM and PI as minor component. CE, TG and C are the dominant classes in the NL profiles (Figure 11 A, C). Lyso-PC and PC were substituted predominantly with 18:1 (Figure 11 E, F). PI contributed 5–10 mol% to the phospholipidome of the apoe−/− and *fads2−/−* x *apoe−/−* double mutants (Figure 11A), which contained the 18:0/20:3-DAG species, but the two main species, surprisingly, were 38:4 (18:0/20:4-PI) and 38:5 (18:1/20:4-PI), present in almost identical concentration in the two genotypes.

Inactivation of the *fads2−/−* locus in the HFHC-fed diet *apoe−/−* and *fads2−/−* x *apoe−/−* double mutant had no impact on the high total C, CE, and TG concentrations in the profile of NL classes of serum Lps (Figure 11C). GC/MS analyses revealed similar fatty acid profiles of total lipids of serum Lps of control (Figure 11H), *fads2−/−* (Figure 11J) *apoe−/−* (Figure 11I) and *fads2−/− x apoe−/−* mice (Figure 11K) on regular and HFHC diet.

### 3.6 HFHC diet does not induce atherosclerosis in PUFA synthesis–deficient *fads2−/−* mice

**Figure 12:**
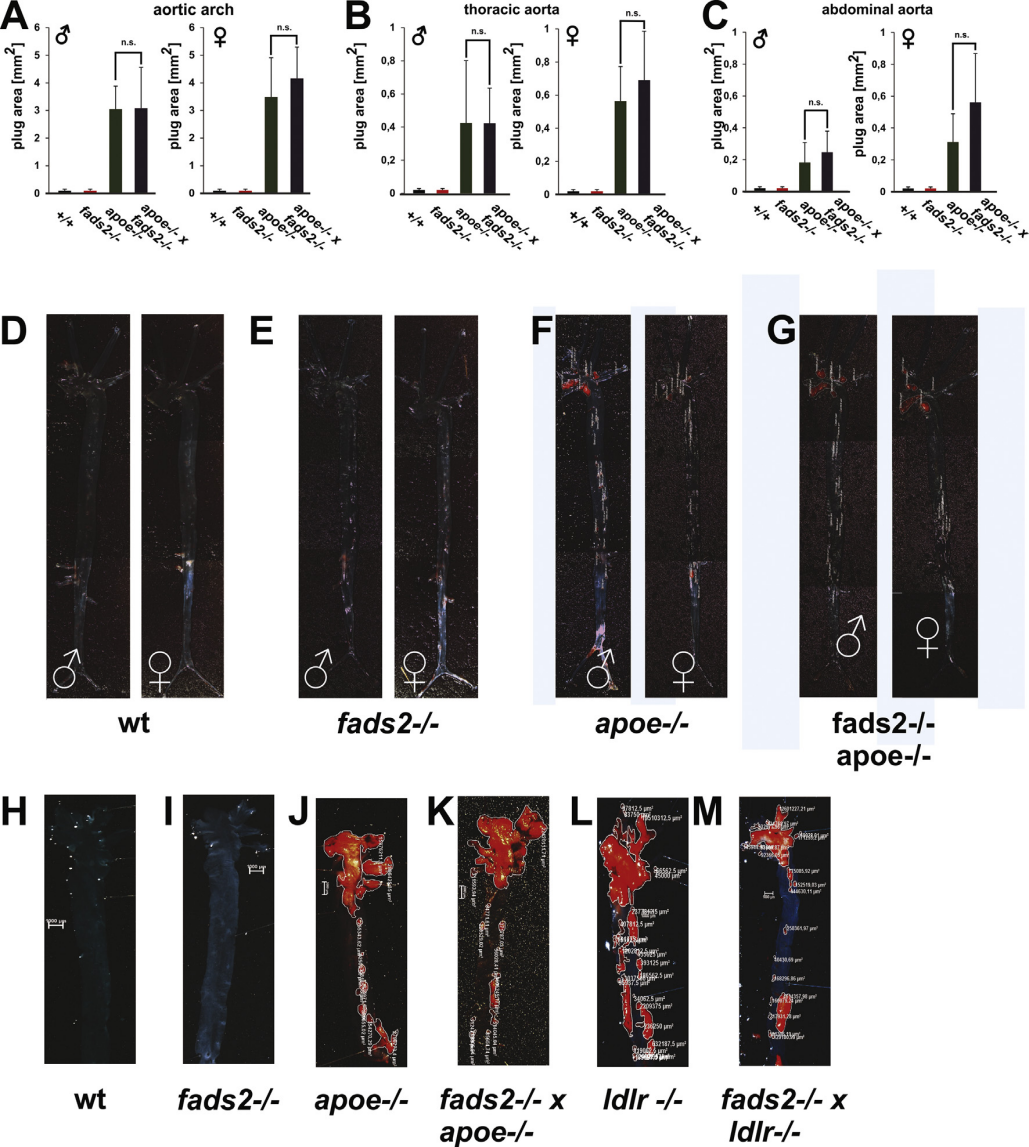
Resistance of PUFA synthesis–deficient *fads2*−/− mice to sustained HFHC diet and HFHCc- (Paigen diet diet) inducing atherosclerosis. Planimetric quantification of oil red–stained atherosclerotic lesions of the (A) aortic arc and (B) thoracic and (C) abdominal aorta in longitudinally opened aortae of age- and gender-matched cohorts of mice (*n* = 10) on a four-month HFHC feeding regime, started after weaning. Paradigmatic en face images of (D) +/+, (E) *fads2*−/−, (F) *apoe*−/− and (G) *fads2*−/− *x apoe*−/− mice. HFHCc diet for 8 months after weaning in cohorts: en face images: (J) +/+, (K) *fads2*−/−, (L) *apoe*−/−, (M) *fads2*−/− *x apoe*−/−, (N) *ldlr*−/− and (O) *fads2*−/− *x ldlr*−/− mice. Planimetry of lesions in (H) aortic arch and (I) thoracic aorta of (J–O), *n* = 5.

Age- and gender-matched +/+ and *fads2*−/−, *apoe*−/− and *fads2*−/− x *apoe*−/− cohorts were fed a PUFA-free HFHC diet for 4 months after weaning. En face images of longitudinally opened, oil red–stained aortae were used for planimetry of atherosclerotic lesions in the aortic arc and thoracic and abdominal aorta, the results of which are presented as bar diagrams (Figure 12A-C).

Atherosclerotic lesions were absent along the entire aortae of +/+ and *fads2*−/− mice (Figure 12 D, E). However, the aortic arc and thoracic and abdominal aorta to the iliac bifurcation of the *apoe*−/− cohort were seeded with plaques (Figure 12F). Inactivation of the fads2 locus in the *apoe*−/− genome changed neither the number nor expansion of thoracic and abdominal lesions in male and female *fads2*−/− x *apoe*−/− double mutant cohorts (Figure 12G).

Sodium cholate–supplemented HFHC diet accelerates massive plaque formation in *apoe*−/− and *ldlr*−/− mice [18].

In another feeding experiment, we fed this diet for 8 months to age- and gender-matched C57BL/6N (Figure 12J), *fads2*−/− (Figure 12K), *apoe*−/− (Figure 12), *fads2*−/− *x apoe*−/− (Figure 12M), *ldlr*−/− (Figure 12N), and *fads2*−/− *x ldlr*−/− mice (Figure 12O). *Fads2*−/− mice developed no atherosclerotic lesions compared to the massive distribution and plaque size along the entire aorta of apoe−/− (Figure 12L) and *ldlr*−/− mice (Figure 12N). Inactivation of the *fads2*−/− locus in the *apoe*−/− and *ldlr*−/− mutants neither reduced nor increased the number and size of atherosclerotic lesions of *fads2*−/− *x apoe*−/− (Figure 12M) and *fads2*−/− *x ldlr*−/− double mutants (Figure12O).

**Figure 13:**
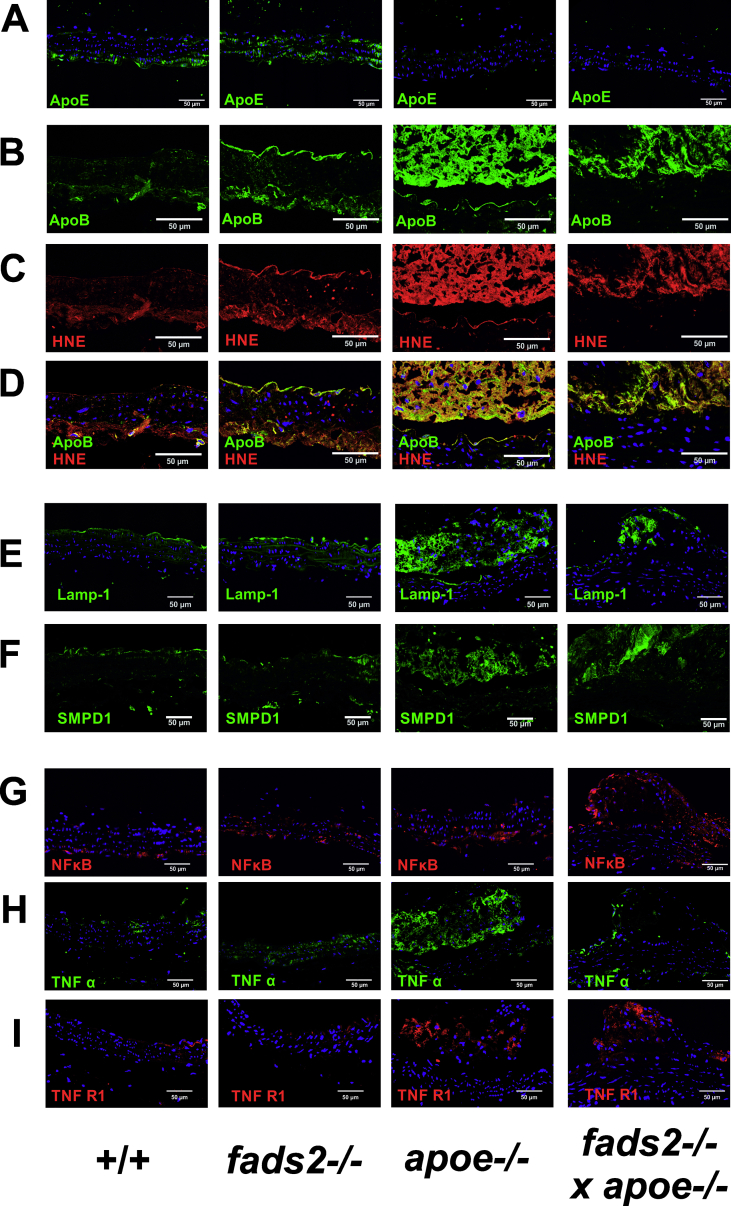
PUFA synthesis deficient fads2−/− mice are resistant to atherosclerotic plaque formation by sustained HFHC diet (4mo). IF-microscopy of sections of vessel wall of aortae of +/+ and *fads2−/−* males *apoe−/−* and *apoe−/− x fads2−/−* aorta was used for probing infiltration of (A) apoE, (B) apoB, (C) HNE and (D) merged ApoB/HNE of (B) and (C), lysosomal markers (D) anti LAMP1- and (F) anti-SMPD1 (acid sphingomyelinase) antibodies, and markers of inflammation (G) anti-NFkB, (H) anti-TNFα- and (I) anti-TNFR1 antibodies. The plaques in the *apoe−/−* and *fads2−/−apoe−/−* aorta sections are partially detached from the vessel wall.

Aortic lesions were further characterized by immuno-histochemical demonstration of infiltrated subendothelial lipoproteins in the arterial wall, probing for apoE and apoB by anti-apoE and anti-apoB antibodies (Figure 13A,B). IHC revealed only minor deposits of apoE in +/+ and fads2−/− aorta, with the expected absence in the *apoe*−/− aorta.

Oxidative stress, manifesting as peroxidation of EFAs and PUFAs, releases highly reactive aldehydes. Trans-4-hydroxy-2-nonenal is a dominant oxidation product of linoleic acid and PUFAs [19,20], which derivatize LDL to “oxidized LDL” (LDLox), an essential factor in the development of atherosclerotic vessel lesions [[21], [22], [23], [24]]. Merged images of sections of aorta vessel walls of +/+ and *fads2*−/− mice stained with anti-HNE and anti apoB antibodies revealed no labelled deposits in lesions, but highlighted HNE-modified apoB in the atherosclerotic plaques of *apoe*−/− and *apoe*−/− *x fads2*−/− aortae after the sustained HFHC feeding period (Figure 13B–D).

### 3.7 PUFA deficiency has no effect on macrophage activation and inflammation in aortic lesions

LDLox uptake by the endothelial lining of the aorta triggers chemotactic engulfment of monocytes and transformation into macrophages (foam cells), visible as fatty streaks. IHC with lysosomal markers LAMP1 and SMPD1 antibodies visualized only low numbers of phagosomes in macrophages within aortic lesions of +/+ and *fads2*−/− mice, but there was an accumulation of lesions of the *apoe*−/− and *fads2*−/− *x apoe*−/− aorta (Figure 13E, F).

We further assessed the contribution of macrophages to the inflammation process by IHC using anti-NFκB, anti-TNFα and anti-TNFR1 antibodies, which did not reveal expression of these markers in apoe−/− mice and revealed less-severe expression in *fads2*−/− *x apoe*−/− double mutants in the aortae of either control or *fads2*−/− male and female mice after the prolonged HFHC feeding period (Figure 13 G-I).

## 4 Discussion

The imbalanced ratio of ω3-and ω6-PUFAs in the current Western diet is regarded as a critical pathogenetic and epigenetic factor of the increasing incidence of metabolic syndrome and associated risk of developing dyslipoproteinemia and atherosclerosis in cardiovascular disease [[2], [3], [4]]. These conditions have been investigated in numerous complex interventional nutritional trials in human and animal models, with contradictory outcome and limited insight into the molecular pathophysiology. There is therefore a current need for unbiased preclinical animal models [3,4].

The current study uses the auxotrophic *fads2*−/− mouse mutant, characterized by the inactivation of Δ6-desaturase, the key enzyme of ω3-and ω6-PUFA synthesis from EFAs linoleic and α-linolenic acid. Bypassing potential confounding factors, this mutant proves congenial for elaborating the structural and metabolic roles of ω3-and ω6-PUFAs and PUFA deficiency [6,21] in C57BL/6 and *fads2*−/− mice in controlled long-term feeding regimens of a) regular chow and b) high fat/high cholesterol (HFHC) PUFA-free diet (“Western diet”). We describe the structural modification of the steady-state lipidomes of membrane lipid bilayers and the metabolic response of a) liver endoplasmic reticulum (ER), the site of NL and PL synthesis in PUFA-deficient *fads2*−/− mice, b) the role of the modified asymmetric ER membrane lipid bilayer as donor of the PL monolayer and NL core of lipid droplets (LD), c) the assembly of the serum lipoprotein (Lp) transport system and d) the development of dyslipoproteinemia and atherosclerosis.

Our study disclosed a stable stoichiometry of PL classes in the ER lipidome of liver of control and *fads2*−/− mice on regular control chow, but extensive structural changes of DAG profiles of individual PL classes of the *fads2*−/− mouse. The entire repertoire of ω3-and ω6-PUFAs in the hydrophobic DAG core, particularly PC, PS, PI and PE of the ER bilayer of liver of the *fads2*−/− offspring of heterozygous foster mothers born with the wt-lipidome, is replaced by the single surrogate 20:3 ^5,11,14^ acid, following a linear regression. We have discovered 20:3 ^5,11,14^ as the end-product of an aberrant biosynthetic pathway of linoleic acid in the *fads2−/−* mouse, and previously demonstrated that Δ8-desaturase is absent and the desaturation of 20:3 5,11,14 to arachidonic acid (20:45,8,11,14) precluded in mammalian tissues [6,25,25].

Our study underscores the pivotal role of the severely modified hydrophobic core structures of the ER lipid bilayer scaffold and perturbation of lipid metabolism of the liver. We concluded that the disrupted divinyl-methane (homo-allylic) rhythm in the 20:3 ^5,11,14^ acid, the sole sn2 substituent of all ω3-and ω6-PUFA across the entire phospholipidome, critically impinges on the spatial arrangement of the DAG core structure and molecular architecture of the lipid bilayer, leading to asymmetry of the liver's ER membranes in the *fads2*−/− mutant. This interpretation is substantiated by comparative monolayer studies of 20:3^5,11,14^ and 20:4^5,8,11,14^ films in the horizontal Langmuir-type surface film balance. Pressure/area (π-A) isotherms revealed equal stability at the film collapse point (pressure 26 mN/m), but considerably different spatial requirements, up to the point of tightest packing, 22.5 Å^2^/molecule 20:4^5,8,11,14^ vs 12 Å^2^/molecule 20:3^5,11,14^ (SI Figure 5).

It is remarkable that only the EFA 18:2, not α-18:3, is utilized as substrate in the synthesis of surrogate structures of the depleted ω3-and ω6-PUFAs by the tetrameric chain elongation complex and trimeric desaturase FADS1 complex in the ER of *fads2*−/− mutants. Our GC/MS analyses of fatty acid methyl-esters and dimethyl-oxazoline (DMOX) derivatives for validation of the double bond positioning in each clearly demonstrated the absence of α-linolenic acid (18:3^9,12,15^) in the neutral- and phospholipidome. The comprehensive analyses revealed that unlike linoleic acid, α-linolenic acid (18:3^9,12,15^) is not further transformed to 20:4^5,11,14,17^ or any other 18:3^9,12,15^ derivative. These finding support earlier observations that mice poorly convert, but preferentially β-oxidize, α-linolenic acid [26,27]**.**

The current concept of membrane lipid bilayer asymmetry views SM, C and PC as abundant PL classes of the luminal leaflet and PS, PI and PE of the cytoplasmic leaflet of the ER membrane [28,29]. Our study provides strong lipid-based structural arguments supporting the concept that the PL monolayer of the cytoplasmic leaflet of the ER-bilayer buds outwardly to form the monomolecular layer wrapping the NL (TG, C and CE) core of lipid droplets. PC, PS, PI and PE represent essential elements of the cytoplasmic leaflet of the ER bilayer and the LD monolayer. Their DAG species are strikingly similar in the cytoplasmic leaflet of ER and the monolayer of LDs of control (+/+) liver and likewise of *fads2*−/− liver, but devoid of PUFAs and substituted by the 20:3^5, 11, 14^ surrogate.

We observed that HFHC diet a) suppresses 20:3^5,11,14^ surrogate synthesis, which includes chain elongation of 18:2 at the tetrameric elongase and a Δ5-desaturase reaction at the trimeric desaturase complex in the ER compartment, and b) surprisingly, strongly inhibits the depletion of all PUFAs in the lipidome of *fads2−/−* liver, documented here in the lipidomes of ER, LD (Figure 5G,H, I, J K,L) and Lp (Figure 10L). The disclosure of the molecular basis of this important metabolic and nutritional interplay awaits future experiments.

Our experiments suggest the fundamental importance of ω3-and ω6-PUFAs as PL constituents of the lipid bilayer structure, the scaffold of enzymes and transcription factor of lipogenesis in liver. Inactivation of the *fads2* locus in the *fads2*−/− mouse perturbs liver lipid metabolism, lipid storage (including processing of inactive precursor proteins of sterol regulatory element-binding protein (SREBP) transcription factors [[30], [31], [34]] and causes dyslipoproteinemia.

Studies in primary rat hepatocytes or in liver have demonstrated that liver X receptor α (LXRα) is not regulated by PUFAs as a ligand, and the suppression of SREBP-1 and its targeted lipogenic genes by unsaturated fatty acids is independent of LXRα [32].

The lipid monolayer is the scaffold of LD-specific proteins, the most abundant of which are Perilipins Plin 1, 2 and 3, equipped with protein binding motifs for targeting, binding and budding of lipid droplets from the ER [33]. As major constituents of the globular surface of LD, they assist DGAT-catalyzed TG synthesis and storage in NDs. ATGL mobilizes lipases and size of droplets and liver steatosis and assembly of lipoproteins.

The perturbed PL monolayer of LD in the liver of *fads2*−/− mice has a profound impact on the accessibility of PAT protein dynamics, mobilization of TG, hydrolysis and synthesis and providing C, CE and TG for loading the Lp transport system. Steady-state expression PLIN2 in ER and LD of *fads2−/−* liver is strongly downregulated and ATGL upregulated (Figure 8A,B).

HFHC diet elevates steady-state concentrations of PLIN1, 2 and 3 in *apoe*−/− liver, which is reduced in *fads2*−/− liver and normalized in the *fads2*−/− *x apoe*−/− double mutant.

PL classes were present in liver ER, LD and Lp-phospholipidomes of control +/+ and *fads2*−/− mice on regular diet in closely similar concentration, but differed severely in their modified DAG structures with different spatial requirements, transmitting a disturbed topology of the polar head group required for the cooperation of Rab5 and phosphatidylinositol (PI) and PI3P and effectors regulating membrane tethering and fusion of LD.

Inactivation of the *fads2* gene locus renders the *fads2*−/− mouse resistant to long-term PUFA-free regular and atherogenic HFHC (Western) diet–induced development of atherosclerotic lesions.

Inactivation of the fads2-locus and PUFAs in the *fads2−/− x apoe−/−* and *fads2−/− x ldlr−/−* double mutants neither accelerated nor retarded the rapid and extensive development of atherosclerotic lesions in the *apoe−/−* and *ldlr−/−* mouse.

The cis-double bond systems of ω3-and ω6-EFAs and PUFAs are chemical targets, highly vulnerable to autoxidation, which releases reactive aldehydes for derivation of proteins, including toxic LDLox, ligand of the endothelial scavenger receptor. Our immune-histochemical results strongly suggest that the absence of PUFAs as highly vulnerable chemical targets of autoxidation prohibits inflammatory responses and formation of atherosclerotic lesions.

## 5 Conclusion and synopsis

The results of this comprehensive dietary study and the previous broad phenotypic characterization of the ω3-and ω6-PUFA synthesis–deficient, unbiased genetic mouse model have widened and focused our molecular insight into the pleiotropic roles of ω3-and ω6-PUFAs. They uncovered the necessity of further scrutinizing molecular studies of the pleiotropic functions of ω3-and ω6-PUFAs as molecular culprits or benefactors during the lifespan in the phase of growth and development [11,12], homeostasis [6,25] and degeneration before being included in legitimate dietary recommendations.

## Author contribution

W.S. supervised the study, designed the experiments and wrote, reviewed and edited the manuscript. E.B. performed and validated experiments. I.S.S. performed and validated experiments and artwork. S.B. performed MS/MS analyses and I.W. performed and validated experiments, reviewed and edited the manuscript.

## Appendix A Supplementary data

Multimedia.Comp 1 Multimedia.Comp 1
