# Supplementary material for: High fat / high cholesterol diet does not provoke atherosclerosis in the ω3-and ω6-polyunsaturated fatty acid synthesis–inactivated Δ6-fatty acid desaturase–deficient mouse
Source: Mol Metab. 2021 Sep 14;54:101335. doi: 10.1016/j.molmet.2021.101335 (PMC8479258; doi:10.1016/j.molmet.2021.101335)
Supplement: Multimedia.Comp 1 [file mmc1.docx]

Supplementary Data

Inactivation of polyunsaturated fatty acid synthesis in the Δ6-fatty acid desaturase deficient (fads2-/-) mouse suppresses atherosclerosis provoked by prolonged high fat / high cholesterol diet

Wilhelm Stoffel*,^1, 2, 3^, Erika Binczek^1^, Inga Schmidt-Soltau^2^, Susanne Brodesser^3^, Ina Wegner^1^

^1^ Laboratory of Molecular Neuroscience, Institute of Biochemistry, University of Cologne, 50931 Cologne, Germany

^2^ Center for Molecular Medicine (CMMC), Faculty of Medicine,

University of Cologne, 50931 Cologne, Germany

^3^ Cluster of Excellence, Cellular Stress Response in Aging-Related Diseases (CECAD), University of Cologne, Cologne, Germany

* Corresponding author

Wilhelm Stoffel, MD, PhD, Laboratory of Molecular Neuroscience, Institute of Biochemistry, University of Cologne, 50931 Cologne Germany

e-mail: [wilhelm.stoffel@uni-koeln.de](mailto:wilhelm.stoffel@uni-koeln.de), phone +49-221-478-6881, fax +49-221-478-6882


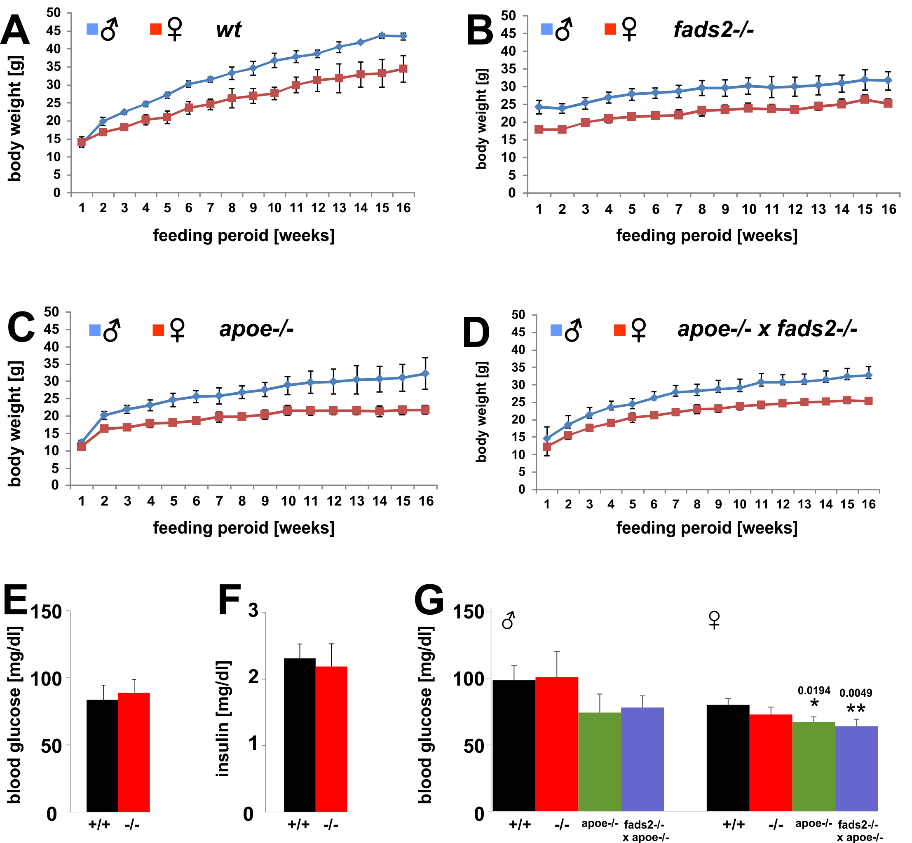


SI Figure 1 Gain of body weight of male and female +/+, fads2-/-, apoe-/- and fads2-/- x apoe-/- mice during 120-days feeding period of HFHC-diet. Male and female cohorts of (A) +/+, (B) fads2-/-, (C) apoe-/- and (D) fads2-/- x apoe-/- mice.

(E) Blood glucose and (F) insulin concentration of +/+ and fads2-/- mice on regular chow, G) Blood glucose concentration of +/+, fads2-/-, apoe-/- and fads2-/- x apoe-/- male and female mice of over-night starved mice on HFHC-diet.


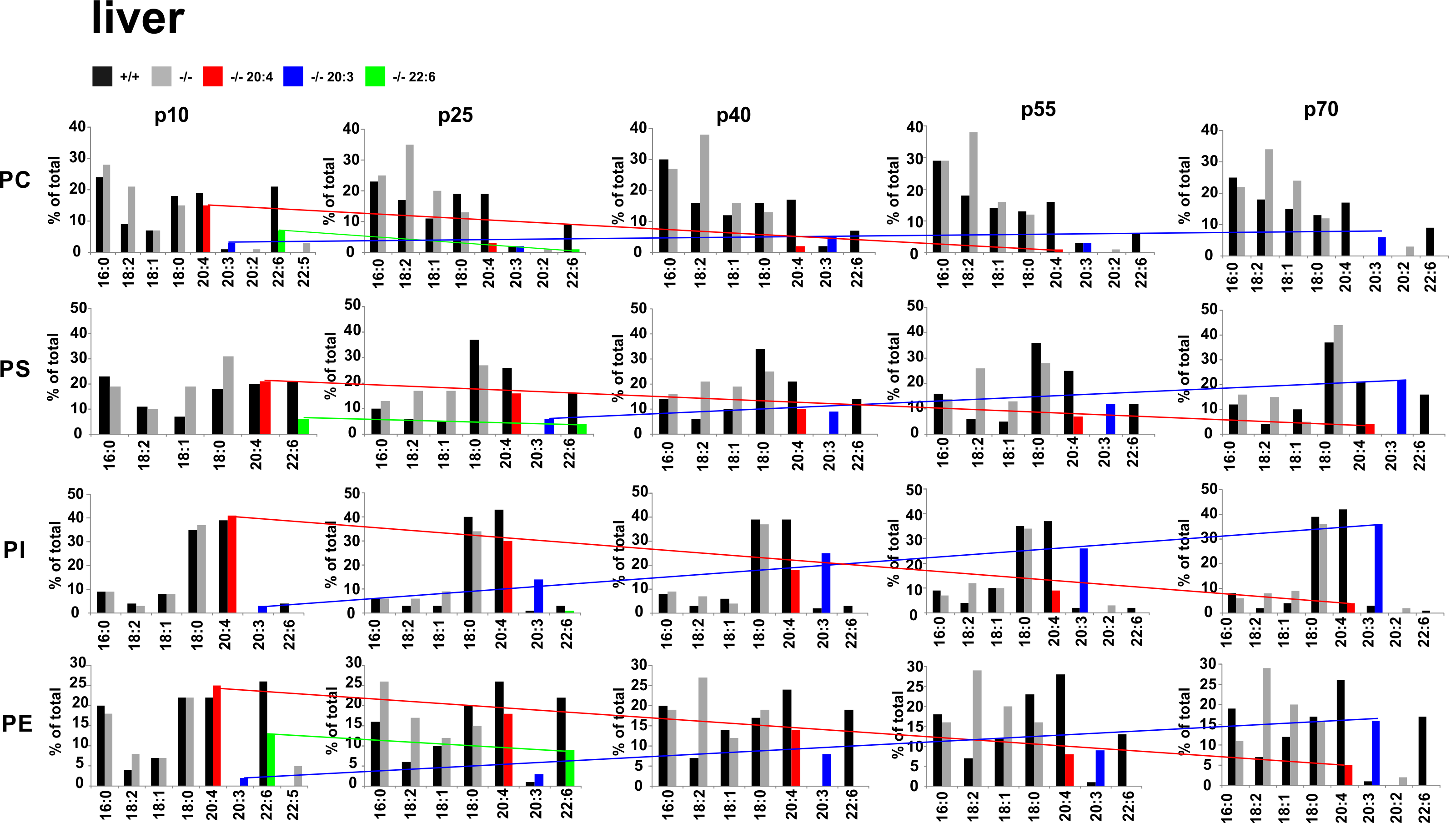


SI Figure 2 Kinetics of the modification of the fatty acid pattern in the phospholipidome of liver of +/+ and fads2-/- mice. HPTLC-separation of PL –classes and densitometric quantification of steady state concentrations of MS/MS-characterized DAG-species of liver phospholipid classes of +/+, fads2-/-, apoe-/- and fads2-/- x apoe-/- mice at p10, p25, p40, p55 and p70.

*Fads2-/-* liver is depleted from ω3-DHA (green bars) in all PL-classes at p30, ω6-AA (red bars) persisted in trace concentrations and 20:3^5,11,14^ (blue bars) linearly increased to the concentration of AA of the respective PL class, particularly in PS, PI and PS in the developing *fads2-/-* mice.


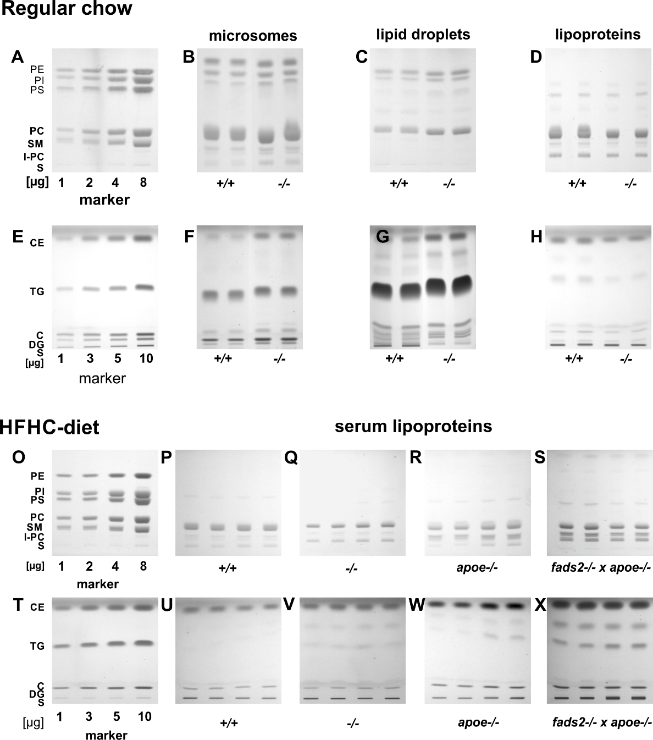
SI Figure 3 Images of densitometric quantification of charred HPTLC-separated PL (B-D) and neutral lipid classes (F-H) in total lipid extracts of liver microsomal fraction, liver lipid droplets and lipoproteins. Quantification PL (P-S and NL (U-X) of serum lipoproteins) after 120 days HFHC-diet of +/+ and fads2-/- apoe-/- and fads2-/- x apoe-/- mice after 120 days normal chow. Lipid extracts of four mice each were pooled for three analyses each. (A, D, O, T) marker lipids.


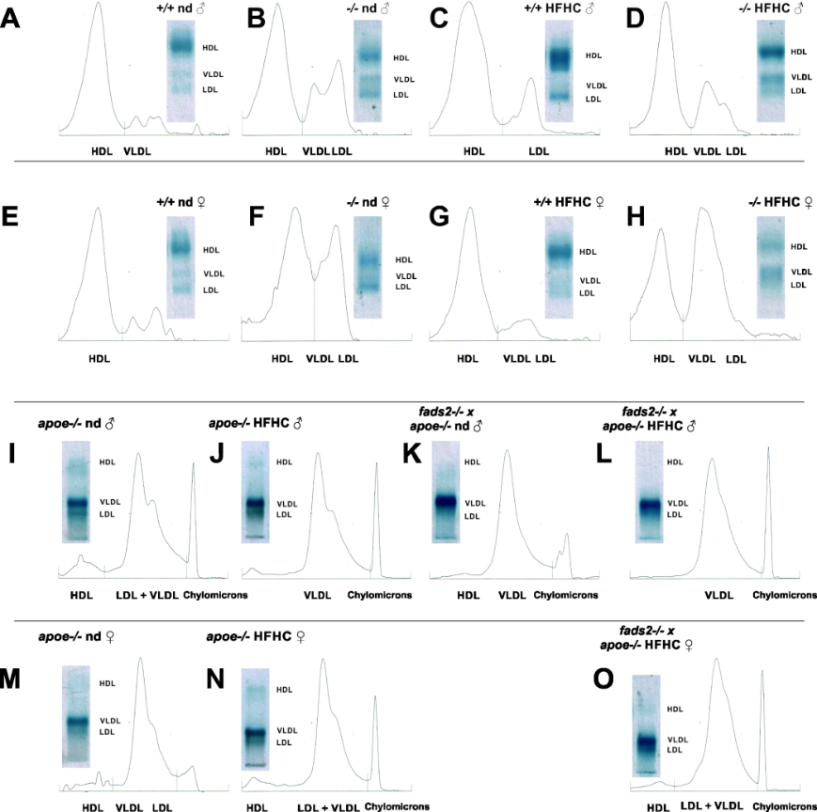
SI Figure 4 HFHC-diet causes severe changes of Lp –profiles of male and female *fads2-/-* mice. Sebia HYDRASYS agarose gel electrophoresis Quantification the serum lipoprotein pattern of adult (4mo) mice on regular chow of (A) male +/+ and (E) female *+/+*, (B) and (F) *fads2-/-,* and (I) and (M) *apoe*-/-,(K) *fads2-/- x apoe-/-* male; on HFHC-diet (C) and (G) +/+ and (D) and (H) *fads2-/-,* (J) and (N) *apoe-/-* and (L) and (O) *fads2-/- x apoe-/-*. Cohort sizes n=3.

π-A- isotherms were recorded continuously and automatically. The teflon coated thermostated trough with the sub-phase was kept at constant temperature of 25°C. Benzene was used as solvent for spreading the monolayer on double distilled water. The synthesis and properties of 20:3^5,11,14^, synthesized in this laboratory, have been described previously [[1](#_ENREF_1)].

Pressure at the collapse point of 20:3^5,11,14^ was 26.00mN/m and the molecular area 12Å^2^ compared to 25.00mN/m and 22.5 Å^2^ of 20:4 ^5,8,11,14^.


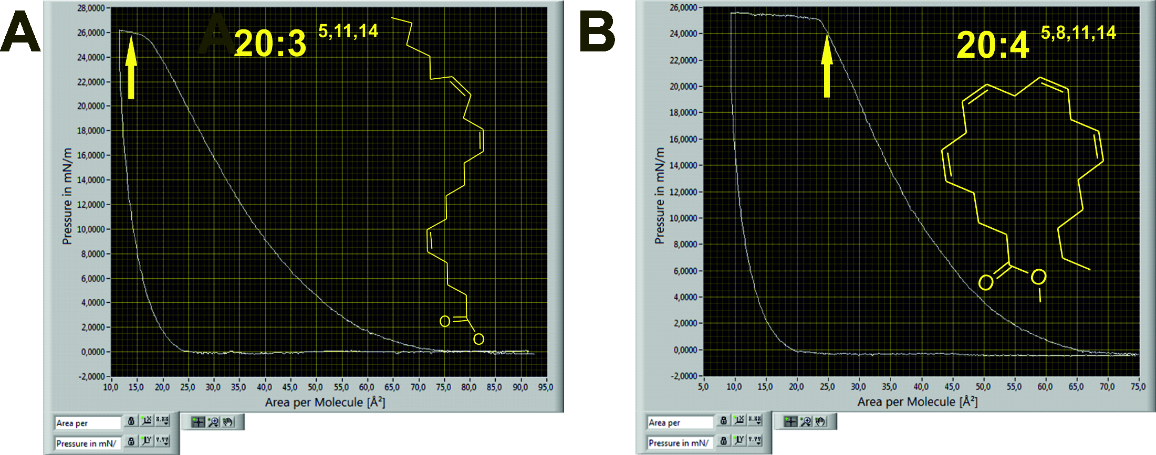
SI Figure 5 25°C π-A- isotherms of 20:3^5,11,14^ and 20:4^5,8,11,14^ .The properties of the monolayers of 20:3^5,11,14^ and 20:4^5,8,11,14^ were studied with the RK1-Standard horizontal Langmuir-type surface film balance with full analog electronic to measure pressure, area, barrier speed, temperature and data recording by computer (Riegler & Kirstein,14467 Potsdam, Germany).

Reference

[1] Hammels, I., Binczek, E., Schmidt-Soltau, I., Jenke, B., Thomas, A., Vogel, M., et al., 2019. Novel CB1-ligands maintain homeostasis of the endocannabinoid-system in omega3- and omega6-long chain-PUFA deficiency. J Lipid Res. 60(8):1396-1409
